# Genetic architecture of seed protein composition in grain amaranth (*Amaranthus hypochondriacus*): a multi-environment genome-wide association study

**DOI:** 10.3389/fnut.2026.1758193

**Published:** 2026-03-10

**Authors:** Rashmi Chauhan, Sharat Prabhakaran, Dinesh Chandra Joshi, Rahul Chandora, J. P. Jaiswal, Dinesh Pandey

**Affiliations:** 1 Department of Molecular Biology & Genetic Engineering, College of Basic Sciences & Humanities, G.B. Pant University of Agriculture and Technology, Pantnagar, Uttarakhand, India; 2 Division of Crop Improvement, ICAR-Vivekananda Institute of Hill Agriculture (Vivekananda Parvatiya Krishi Anusandhan Sansthan), Almora, Uttarakhand, India; 3 ICAR-NBPGR (Regional Station), Shimla, Himachal Pradesh, India; 4 Department of Genetics & Plant Breeding, College of Agriculture, G.B. Pant University of Agriculture and Technology, Pantnagar, Uttarakhand, India

**Keywords:** albumin, amaranth, candidate genes, glutelin, GWAS, marker trait association, pseudocereal, seed protein fraction

## Abstract

**Introduction:**

Grain amaranth (*Amaranthus hypochondriacus*), a nutrient-rich pseudocereal, holds immense potential for protein biofortification, yet the genetic architecture of its seed protein fraction composition remains uncharacterized.

**Methods:**

To dissect this complex trait, we conducted a multi-environment genome-wide association study (GWAS) on a diversity panel of 192 accessions for albumin, globulin, glutelin, prolamin, and total protein content. Significant phenotypic variation was observed for all fractions, with high broad-sense heritability (H^2^ ≥ 0.91) for albumin and total protein, indicating strong genetic control and high selection potential. A multi-locus GWAS using 41,931 SNPs identified 356 significant marker-trait associations (MTAs).

**Results:**

Crucially, filtering for cross-environmental stability revealed 17 robust MTAs for total protein (6), albumin (5), glutelin (5), and globulin (1), while prolamin content was governed by environment-specific loci. Strikingly, candidate gene analysis of these stable loci revealed that natural variation is predominantly controlled by trans-regulatory mechanisms rather than by cis-variation in the storage structural genes themselves. Key regulatory hubs were identified, implicating the abscisic acid (ABA) biosynthesis and signaling pathway in glutelin accumulation and endoplasmic reticulum (ER) protein folding capacity in limiting globulin content.

**Discussion:**

This work establishes the first comprehensive genomic framework for seed protein composition in amaranth, providing environmentally stable markers and validated biological pathways to accelerate the development of nutritionally enhanced cultivars through marker-assisted selection.

## 1 Introduction

The global food system faces the dual challenge of nourishing a growing population while minimizing its environmental footprint. This has intensified the search for sustainable, high-quality plant-based protein sources to mitigate the impacts of traditional agriculture and address widespread protein-energy malnutrition. A critical strategy for achieving this is dietary diversification, moving beyond a heavy reliance on a few major cereals toward the integration of nutrient-dense, climate-resilient crops (1). Pseudocereals, such as quinoa, buckwheat, and amaranth, are at the forefront of this movement, increasingly recognized for their exceptional nutritional profiles, gluten-free properties, and robust agronomic performance.

Among these, grain amaranth (*Amaranthus* spp.) stands out as a “millennium crop,” uniquely equipped to thrive in future agricultural landscapes (2, 3). Its agronomic tenacity, including notable tolerance to drought, heat, and soil salinity, allows for cultivation on marginal lands with fewer inputs, making it a cornerstone for developing sustainable farming systems (4). Nutritionally, amaranth grain offers a protein content of 12.5 to 22.5%, but its true value lies in its superior protein quality. The amino acid composition of amaranth protein closely aligns with the standards recommended by the WHO/FAO, featuring a rich supply of lysine and sulfur-containing amino acids, which are critically deficient in most staple cereals (2, 5). This makes amaranth an invaluable resource for enhancing food quality and a safe, nutritious option for individuals with celiac disease.

This superior nutritional profile is a direct result of the unique composition of its seed protein fractions. In stark contrast to cereals, which are dominated by nutritionally poor prolamins, amaranth seeds are predominantly composed of highly digestible albumins (19–45%) and globulins (16–35%), supplemented by a significant glutelin fraction (22–41%) (6, 7). The albumin fraction, a reservoir of essential amino acids, contains proteins of such high biological value that they serve as a genetic resource for global biofortification. While these Osborne fractions are biochemically defined by differential solubility and contain diverse protein types, in amaranth seeds they are quantitatively dominated by proteins serving storage and reserve functions. The albumin fraction (water-soluble) includes storage albumins alongside metabolic enzymes and cytoplasmic proteins; the globulin fraction (salt-soluble) is enriched in 7S and 11S storage globulins; the glutelin fraction (alkali-soluble) contains 11S-type proteins homologous to cereal glutelins; and the prolamin fraction (alcohol-soluble) is minimal compared to cereals. The gene for one such albumin, AmA1, has been successfully expressed in crops like potato and wheat, acting as a proof-of-concept by substantially increasing their total protein and essential amino acid levels, underscoring the transformative potential held within the amaranth genome (8, 9).

Despite this immense potential, amaranth remains an underutilized crop, and the genetic architecture governing its valuable protein composition is largely unknown. While classical breeding has made some progress, its efficiency is often limited by long selection cycles and the complex, polygenic nature of nutritional traits. The advent of modern genomics offers a powerful alternative to accelerate crop improvement. The recent availability of high-quality reference genomes for key *Amaranthus* species, including *A. hypochondriacus* (10, 11), has created an unprecedented opportunity for high-resolution genetic dissection. Genome-Wide Association Studies (GWAS) have emerged as a premier tool for this purpose, leveraging natural genetic diversity to pinpoint loci controlling complex traits with greater precision than traditional QTL mapping. This approach has been highly successful for identifying loci associated with protein content in major crops like wheat (12), pea (13), and rice (14). While initial genomic studies in amaranth have successfully explored genetic diversity and morphological traits (15), no comprehensive GWAS has been performed to specifically unravel the genetic basis of its distinct seed protein compositions.

A further layer of complexity is added by genotype × environment (G × E) interactions, where the performance of a genetic locus can vary significantly across different growing conditions. Breeding programs based on single-environment data risk developing cultivars that are not widely adapted. Consequently, multi-environment trials are indispensable for identifying environmentally stable genetic loci that exert consistent effects. Such stable loci are the most valuable assets for developing broadly adapted, nutritionally superior cultivars. By pairing a multi-environment experimental design with robust multi-locus GWAS models, we can identify high-confidence marker-trait associations (MTAs) that are primed for deployment in genomic-assisted breeding.

In the present study, we aimed to bridge this critical knowledge gap by conducting the first comprehensive genetic dissection of accumulation of seed protein fractions in grain amaranth. Our objectives were to: (i) characterize the phenotypic variation and estimate key genetic parameters for albumin, globulin, glutelin, and prolamin content in a global diversity panel of 192 *A. hypochondriacus* accessions evaluated across two contrasting environments; (ii) identify novel and environmentally stable MTAs associated with each protein fraction using a multi-locus GWAS approach; and (iii) nominate putative candidate genes within these associated genomic regions to illuminate the molecular mechanisms regulating seed protein accumulation. This research establishes a foundational genomic framework to guide the precision breeding of amaranth for enhanced protein quality, providing tangible tools to develop superior cultivars capable of combating malnutrition.

## 2 Materials and methods

### 2.1 Germplasm panel and genotypic dataset

This investigation was conducted on a diversity panel comprising 192 accessions of *Amaranthus hypochondriacus*. This panel, previously described by Chauhan et al. (16), represents global genetic diversity with accessions originating from Asia, Europe, North America, and Africa. The complete germplasm characteristics table providing detailed passport information for all 192 accessions have been provided in Supplementary Table S1. We leveraged the pre-existing, high-density genotypic dataset for this panel, which consists of 41,931 high-quality single nucleotide polymorphisms (SNPs) generated via Genotyping-by-Sequencing (GBS). Genomic DNA from 192 *Amaranthus hypochondriacus* accessions was subjected to genotyping-by-sequencing (GBS) analysis following established protocols (16). Briefly, DNA samples (5 μg) were digested with ApeKI restriction enzyme, ligated with barcoded adapters, and the multiplexed library was sequenced on Illumina HiSeq X10 platform. Raw reads were demultiplexed using FASTX Toolkit (v0.0.13), quality-filtered with FastQC (v0.11.5), trimmed using Trim Galore (v0.6.2), and aligned to the *A. hypochondriacus* v2.1 reference genome (10) using BWA-MEM (v0.7.5). SNP calling was performed using the GATK pipeline (v3.6), and variants were filtered using VCFtools (v0.1.17) with thresholds of minor allele frequency > 5% and < 20% missing data, yielding 41,931 high-quality SNPs. All bioinformatics analyses were conducted on a workstation with 250 GB RAM and 32-core Intel Xeon processor. All raw sequencing data are accessible through the NCBI Sequence Read Archive under accession number PRJNA1208757.

### 2.2 Multi-environment field evaluation

To assess phenotypic performance across different environmental conditions, the 192-accession panel was evaluated in two contrasting agro-ecological zones within Uttarakhand, India. The first site, ICAR-Vivekananda Parvatiya Krishi Anusandhan Sansthan (VPKAS) in Almora, represents a high-altitude, hilly ecosystem (1,650 m asl). The second site, the Pantnagar Center for Plant Genetic Resources (PCPGR) in Pantnagar, is located in the subtropical Tarai foothills (243.8 m asl). The field evaluations were conducted during the 2021 growing season (June–November) at both locations. The geographical and environment conditions are given in Supplementary material 2.

The experimental layout at each location was an alpha-lattice design with two replications. Each accession was planted in plots composed of two 3-meter rows with 45 cm inter-row spacing. Seedlings were thinned approximately 15–25 days after planting to achieve a uniform stand density. Standard agronomic practices for grain amaranth cultivation were followed throughout the growing season at both locations.

### 2.3 Seed protein fractions profiling

#### 2.3.1 Sample preparation and fractionation

Post-maturity, representative seed samples were obtained by harvesting and pooling three central plants from each plot. The seeds were threshed in bulk, dried, and stored at 4 °C. For biochemical analysis, 5 g of seeds from each accession were milled into a fine flour and passed through an 80-mesh sieve. The flour was stored at −20 °C prior to protein extraction.

The seed flour was subjected to sequential protein extraction to isolate four solubility-based fractions: albumin, globulin, prolamin, and glutelin. This procedure was based on the classical Osborne (55) classification, following a modified protocol adapted from Landry and Moureaux (17). Briefly, 0.1 g of flour was sequentially extracted three times with each of the following solvents: 10 mM Tris–HCl buffer (pH 7.5) for albumins; 1 M NaCl for globulins; 60% n-propanol with 1 mM EDTA-2Na for prolamins; and finally, 0.05 M NaOH for glutelins. Between each solvent extraction, the samples were agitated for 2 h at room temperature and then centrifuged at 12,000 rpm for 15 min at 4 °C. Pellets were washed twice with deionized water between sequential extractions to prevent cross-contamination of protein fractions. The respective supernatants for each fraction were collected and stored at −20 °C.

#### 2.3.2 Protein quantification

The protein concentration in each of the four extracted fractions was determined spectrophotometrically using the Bradford (18) dye-binding assay. Bradford reagent was prepared by dissolving 100 mg of Coomassie Brilliant Blue G-250 in 50 mL of 95% ethanol, followed by addition of 100 mL of 85% (w/v) phosphoric acid, with the final volume adjusted to 1 L. Bovine serum albumin (BSA) was used to generate a standard curve (5–100 μg/mL) for quantification. Sample aliquots (300 μL) were mixed with 3 mL of Bradford reagent, incubated for 15 min, and absorbance was measured at 595 nm. All measurements were conducted in triplicate. The average protein content for each fraction was calculated and used as the phenotypic value for subsequent analysis.

### 2.4 Statistical modeling and genome-wide association mapping

#### 2.4.1 Phenotypic data analysis

We conducted two complementary statistical analyses with distinct objectives:

1. Environment-specific analysis for GWAS: To identify marker-trait associations, we calculated environment-specific Best Linear Unbiased Predictions (BLUPs) for each accession within each site (Pantnagar and Almora) separately. For each environment, the model TraitValue ~ (1|Taxa) + (1|Rep) was fitted using the lme4 package (19) in R (20). These within-environment BLUPs served as the phenotypic input for separate genome-wide association studies at each location.
2. Combined-environment analysis for genetic parameters: To estimate variance components and genetic parameters, we fitted the full mixed model: TraitValue ~ Env + (1|Taxa) + (1|Taxa: Env) + (1|Rep: Env). In this model, environment (Env) was treated as a fixed effect, while genotype (Taxa), genotype-by-environment interaction (Taxa: Env), and replication within environment (Rep: Env) were treated as random effects. *p*-values for fixed and random effects were estimated using the lmerTest package (21). Treatment of G × E as random effect was justified because our objective was to partition phenotypic variance and estimate genetic parameters (heritability, genetic advance) that enable inferences about the broader population of amaranth genotypes. Variance components (σ^2^G, σ^2^GE, σ^2^ε) derived from this model were used to calculate: - Broad-sense heritability (H^2^) = σ^2^G / (σ^2^G + σ^2^GE/e + σ^2^ε/re), where e = number of environments, r = number of replications, Genotypic coefficient of variation (GCV) = (√σ^2^G / mean) × 100, Phenotypic coefficient of variation (PCV) = (√σ^2^P / mean) × 100, where σ^2^P = σ^2^G + σ^2^GE/e + σ^2^ε/re - Genetic advance (GA) = k × √σ^2^G × H^2^, where k = 2.06 (selection differential at 5% selection intensity) and Genetic advance as percentage of mean = (GA / mean) × 100.

#### 2.4.2 Association mapping

Genome-wide association studies were conducted using the mrMLM v4.0.2 software package (22), an integrated R platform specifically designed for multi-locus association mapping. This analytical framework implements six distinct multi-locus GWAS methods, each with unique statistical properties: (1) mrMLM (multi-locus random-SNP-effect mixed linear model), which uses a two-stage approach where potentially associated markers are first selected then fitted simultaneously in a multi-locus model with effects estimated by empirical Bayes; (2) FASTmrMLM (fast multi-locus random-SNP-effect mixed linear model), an accelerated version of mrMLM with improved computational efficiency; (3) FASTmrEMMA (fast multi-locus efficient mixed-model association), which combines the EMMA algorithm with multi-locus modeling; (4) pLARmEB (integration of least angle regression with empirical Bayes), which uses least angle regression to select potentially associated markers under polygenic background control; (5) pKWmEB (integration of Kruskal-Wallis test with empirical Bayes under polygenic background control), which employs non-parametric testing followed by empirical Bayes estimation; and (6) ISIS EM-BLASSO (iterative sure independence screening expectation–maximization and Bayesian least absolute shrinkage and selection operator), which combines feature screening with Bayesian variable selection. The environment-specific BLUPs calculated separately for Pantnagar and Almora served as input phenotypes for independent GWAS analyses at each location.

To control for false positive associations arising from population stratification and cryptic relatedness, both kinship (K) and population structure (Q) matrices were incorporated as covariates in all association models. The kinship matrix was calculated using the EMMA (Efficient Mixed-Model Association) algorithm as implemented within the mrMLM platform, quantifying genetic relatedness between all pairs of accessions based on genome-wide marker information. The population structure matrix was generated from the first 5 principal components derived from genome-wide SNP data. A critical logarithm of the odds (LOD) threshold of ≥3.0 was consistently applied across all six methods to declare statistically significant marker-trait associations (MTAs). Independent association mapping analyses were conducted for each protein fraction at each experimental location, and MTAs were classified as environmentally stable only when significant associations (LOD ≥ 3.0) were detected for the same SNP-trait combination in both Pantnagar and Almora environments using at least one of the six GWAS methods, providing stringent cross-environmental validation that substantially increases confidence in the identified associations.

### 2.5 Identification of putative candidate genes

Significant MTAs were further investigated to identify putative candidate genes involved in seed protein regulation. A search window of ±250 kb was established around each significant MTA, based on the average LD decay distance (r^2^ declining to 0.2) estimated for this same amaranth diversity panel (16). Briefly, LD decay was calculated using squared correlation coefficients (r^2^) between all pairs of SNPs within 500 kb physical distance across all 16 scaffolds. The population-wide LD decayed to background levels (r^2^ < 0.2) at approximately 220–280 kb (mean = 250 kb), which defined our candidate gene search window. The LD decay plots showing LD decay plot indicating the ~250 kb threshold corresponding to r^2^ = 0.2, for the diversity panel is shown in Supplementary material 2.

## 3 Results

### 3.1 Phenotypic variations, descriptive statistics and distribution patterns in seed protein fractions in *Amaranthus hypochondriacus*

**Figure 1:**
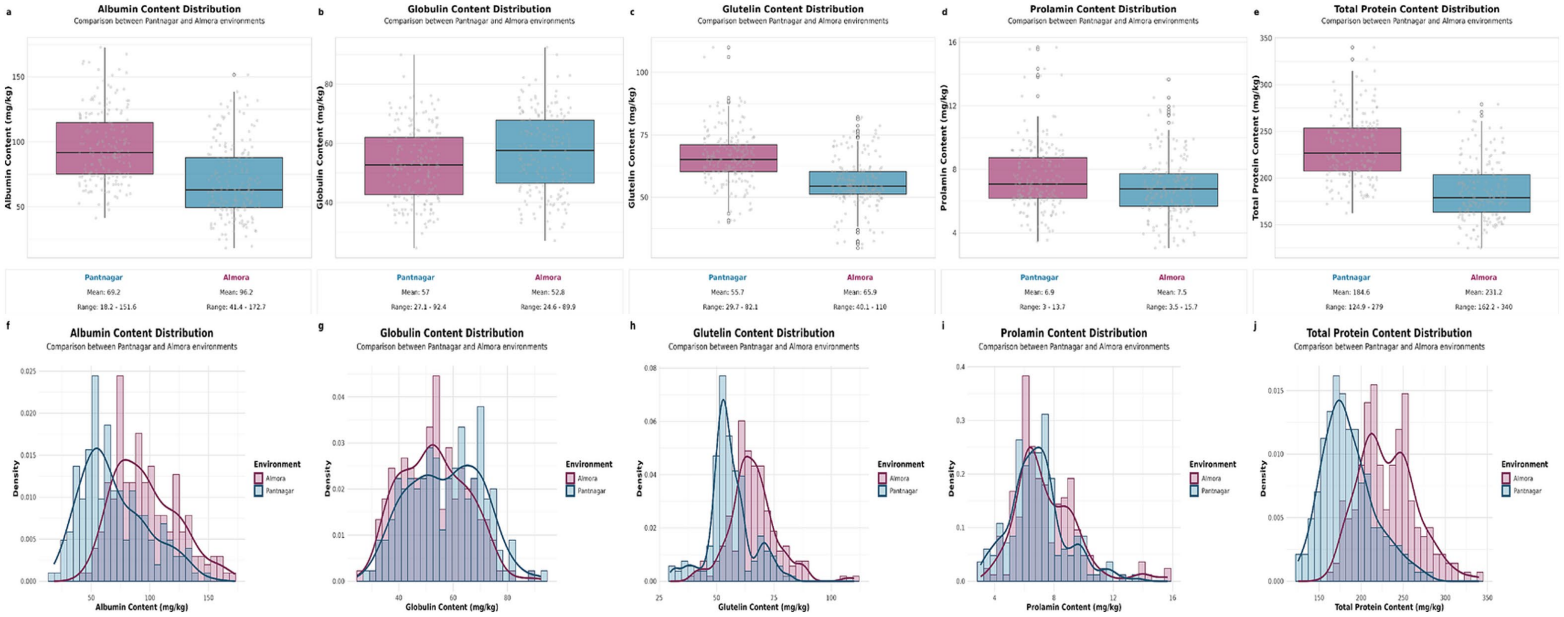
Comparative analysis of protein fraction content in grains *Amaranthus hypochondriacus* germplasm across Pantnagar and Almora environments. Distribution and variation of protein fractions in amaranth germplasm evaluated across two contrasting environments. **(a–e)** Boxplots showing the distribution of albumin, globulin, glutelin, prolamin, and total protein content (mg/kg) for Pantnagar (blue) and Almora (magenta) environments. Box boundaries represent the first and third quartiles, the horizontal line indicates the median, and individual data points are shown as jitters. **(f–j)** Frequency distribution histograms overlaid with density curves for the corresponding protein fractions, illustrating the distributional patterns of each protein type across both environments. Mean values and ranges for each environment are displayed beneath the boxplots. Data represent measurements from 192 *A. hypochondriacus* accessions.

Analysis of 192 grain amaranth accessions evaluated across two distinct environments revealed considerable variation in seed protein fraction composition (Figure 1). All five traits—albumin, glutelin, globulin, prolamin, and total protein—displayed continuous variation with near-normal distributions, characteristic of quantitative traits under polygenic control.

Albumin content demonstrated substantial environmental plasticity, with population means shifting from 69.24 g/kg in environment 1 (Pantnagar) to 96.22 g/kg in environment 2 (Almora)—a 39% increase. The range of variation was considerable in both environments: 16.54–156.76 g/kg in Pantnagar and 39.19–173.13 g/kg in Almora. Despite this wide range, the coefficient of variation decreased from 41.46% in Pantnagar to 28.86% in Almora, suggesting more uniform protein synthesis under Almora conditions. The median values (64.72 g/kg in Pantnagar; 94.85 g/kg in Almora) closely approximated the means, with slight positive skewness (0.61 in Pantnagar; 0.52 in Almora) indicating a few accessions with exceptionally high albumin accumulation. Negative kurtosis values (−0.29 in Pantnagar; −0.32 in Almora) revealed platykurtic distributions with fewer extreme values than expected under normality.

Glutelin, representing the major protein fraction, showed more stable means across environments: 55.69 g/kg in Pantnagar and 65.52 g/kg in Almora—an 18% increase. This fraction exhibited the lowest coefficient of variation among all proteins (18.59% in Pantnagar; 16.47% in Almora), indicating relatively consistent accumulation patterns across genotypes. The phenotypic range spanned 26.92–90.42 g/kg in Pantnagar and 34.82–94.86 g/kg in Almora. Median values (54.77 g/kg in Pantnagar; 65.05 g/kg in Almora) aligned closely with means, and near-zero skewness values (0.14 in Pantnagar; 0.03 in Almora) confirmed nearly symmetrical distributions. The slightly positive kurtosis (0.31 in Pantnagar; 0.22 in Almora) suggested marginally leptokurtic distributions with mild concentration around the mean.

Globulin content ranged from 19.10–95.98 g/kg in Pantnagar and 22.94–106.54 g/kg in Almora, with means of 52.82 and 61.64 g/kg respectively—a 17% environmental increase. This fraction showed moderate variability (CV: 24.29% in Pantnagar; 25.46% in Almora) that remained consistent across environments. The interquartile ranges (18.40 g/kg in Pantnagar; 22.32 g/kg in Almora) captured the central 50% of observations, with minimal skewness (0.10 in Pantnagar; 0.11 in Almora) confirming symmetrical distributions. Negative kurtosis values (−0.40 in Pantnagar; −0.35 in Almora) indicated flatter distributions relative to the normal curve, suggesting diverse globulin accumulation strategies across the germplasm panel.

Prolamin, though the least abundant fraction, displayed the highest relative variation. Mean values were 6.90 g/kg in Pantnagar and 7.47 g/kg in Almora, with remarkably high coefficients of variation (40.05% in Pantnagar; 38.40% in Almora)—nearly double that of other fractions. The ranges (0.26–16.36 g/kg in Pantnagar; 0.57–16.90 g/kg in Almora) revealed some accessions with minimal prolamin while others accumulated substantial amounts. Medians (6.74 g/kg in Pantnagar; 7.29 g/kg in Almora) fell below means, with positive skewness (0.28 in Pantnagar; 0.46 in Almora) confirming right-tailed distributions with several high-prolamin genotypes. The kurtosis shifts from slightly negative in Pantnagar (−0.03) to positive in Almora (0.39) suggests environmental effects on the distribution shape, with Almora promoting more extreme prolamin accumulation in responsive genotypes.

Total protein content integrated all fractions, showing means of 186.59 g/kg in Pantnagar and 231.19 g/kg in Almora—a substantial 24% increase attributable to favorable Almora conditions. Despite this environmental effect, the coefficient of variation remained relatively low (17.44% in Pantnagar; 14.64% in Almora), lower than individual fractions, indicating compensatory mechanisms among protein components. The ranges (121.04–284.14 g/kg in Pantnagar; 162.22–342.08 g/kg in Almora) demonstrated 2.3-fold and 2.1-fold variation, respectively. Moderate positive skewness (0.53 in Pantnagar; 0.48 in Almora) and near-zero kurtosis values (−0.06 in Pantnagar; −0.04 in Almora) characterized nearly normal distributions centered around the means, with medians (182.08 g/kg in Pantnagar; 228.34 g/kg in Almora) slightly below means.

The interquartile ranges provided insights into central tendency dispersion: albumin showed the widest IQR (40.97 g/kg in Pantnagar; 40.94 g/kg in Almora), remarkably consistent across environments despite mean shifts. Total protein displayed similar IQRs (41.91 g/kg in Pantnagar; 44.67 g/kg in Almora), while globulin showed moderate values (18.40 g/kg in Pantnagar; 22.32 g/kg in Almora). Glutelin exhibited the narrowest IQRs (11.05 g/kg in Pantnagar; 13.34 g/kg in Almora), reinforcing its role as a stably accumulated storage protein. Prolamin IQRs (3.61 g/kg in Pantnagar; 3.47 g/kg in Almora) were smallest in absolute terms but largest relative to their means, consistent with high coefficients of variation. Standard errors (SE) were uniformly low across all traits, ranging from 0.14–1.73 g/kg, confirming measurement precision and adequate replication. The consistency of SE values between environments validates the reliability of phenotyping protocols across locations. The complete concentrations of the seed protein fractions among the 192 accessions acroos both loactaions have been provided in Supplementary material 4.

Analysis of variance from the linear mixed model revealed significant effects of genotype and environment for all five seed protein fractions traits, with trait-specific patterns of genotype-by-environment (G × E) interaction (Table 1). Genotypic effects were highly significant for all traits (*p* < 0.001), confirming the presence of substantial genetic variation in the diversity panel for protein composition. Environment had highly significant effects on albumin, glutelin, and total protein (*p* < 0.001), and significant effects on globulin and prolamin (*p* < 0.01), consistent with the substantial phenotypic differences observed between Pantnagar and Almora where mean values increased by 18–39% in the Almora environment compared to Pantnagar (Figure 1).

**Table 1:** Analysis of variance for seed protein content in 192 accessions of *Amaranthus hypochondriacus* across locations.

Source of variation	Albumin	Globulin	Glutelin	Prolamin	Total protein
Environment	***	**	***	**	***
Genotype	***	***	***	***	***
G × E	***	***	**	*	***

***p < 0.001; **p < 0.01; *p < 0.05. *p*-values determined from likelihood ratio tests in linear mixed model analysis.

Critically, G × E interaction was significant for all protein fractions, though with varying magnitudes: highly significant for albumin, globulin, and total protein (*p* < 0.001), significant for glutelin (*p* < 0.01), and significant for prolamin (*p* < 0.05). The universal presence of significant G × E interaction, at different statistical levels, justified our analytical strategy of conducting separate GWAS for each environment followed by stringent cross-environmental validation to identify stable MTAs. This approach allows differentiation between environment-specific genetic effects that manifest only under particular growing conditions and truly stable associations that maintain their effects across contrasting environments. The stable MTAs identified through this cross-environmental validation represent the most valuable targets for marker-assisted breeding programs, as they confer consistent genetic effects across diverse production environments.

### 3.2 Genetic parameters of seed protein content in grain amaranth

Genetic parameter analysis revealed substantial variation in heritability, genetic advance, and variance components across the five seed protein fractions (Table 2).

**Table 2:** Genetic parameters for seed protein content in *A. hypochondriacus*.

Parameter	Albumin	Globulin	Glutelin	Prolamin	Total protein
Heritability (H^2^)	0.933	0.732	0.873	0.523	0.910
Genetic Advance (GA)	50.88	15.74	15.85	1.41	55.96
GA as % of Mean	60.78	27.51	26.07	19.59	26.79
GCV (%)	31.62	18.24	14.49	18.19	14.30
PCV (%)	32.74	21.31	15.51	25.15	14.99
Reliability Ratio	0.972	0.863	0.775	0.354	0.961
Coefficient of Determination (R^2^)	0.853	0.529	0.633	0.215	0.807

Broad-sense heritability ranged from 0.523 to 0.933 across protein fractions. Albumin exhibited the highest heritability (H^2^ = 0.933), followed by total protein (H^2^ = 0.910) and glutelin (H^2^ = 0.873). Globulin showed intermediate heritability (H^2^ = 0.732), while prolamin displayed the lowest heritability (H^2^ = 0.523). Expected genetic advance under selection varied substantially. Total protein showed the largest absolute genetic advance (GA = 55.96 units), followed by albumin (GA = 50.88 units). Glutelin and globulin exhibited moderate genetic advances (GA = 15.85 and 15.74 units, respectively), while prolamin showed minimal genetic advance (GA = 1.41 units). When expressed as percentage of trait mean, albumin demonstrated the highest relative genetic advance (60.78%), followed by globulin (27.51%), total protein (26.79%), glutelin (26.07%), and prolamin (19.59%).

Genotypic coefficient of variation (GCV) ranged from 14.30 to 31.62%. Albumin exhibited the highest GCV (31.62%), substantially exceeding other fractions. Globulin and prolamin showed intermediate GCV values (18.24 and 18.19%, respectively), while glutelin and total protein displayed the lowest GCV (14.49 and 14.30%, respectively). Phenotypic coefficient of variation (PCV) followed similar patterns but with larger values, ranging from 14.99 to 32.74%. The ratio of GCV to PCV indicated the proportion of phenotypic variation attributable to genetic factors.

### 3.3 Genome-wide association analysis of seed protein content in grain amaranth

**Figure 2:**
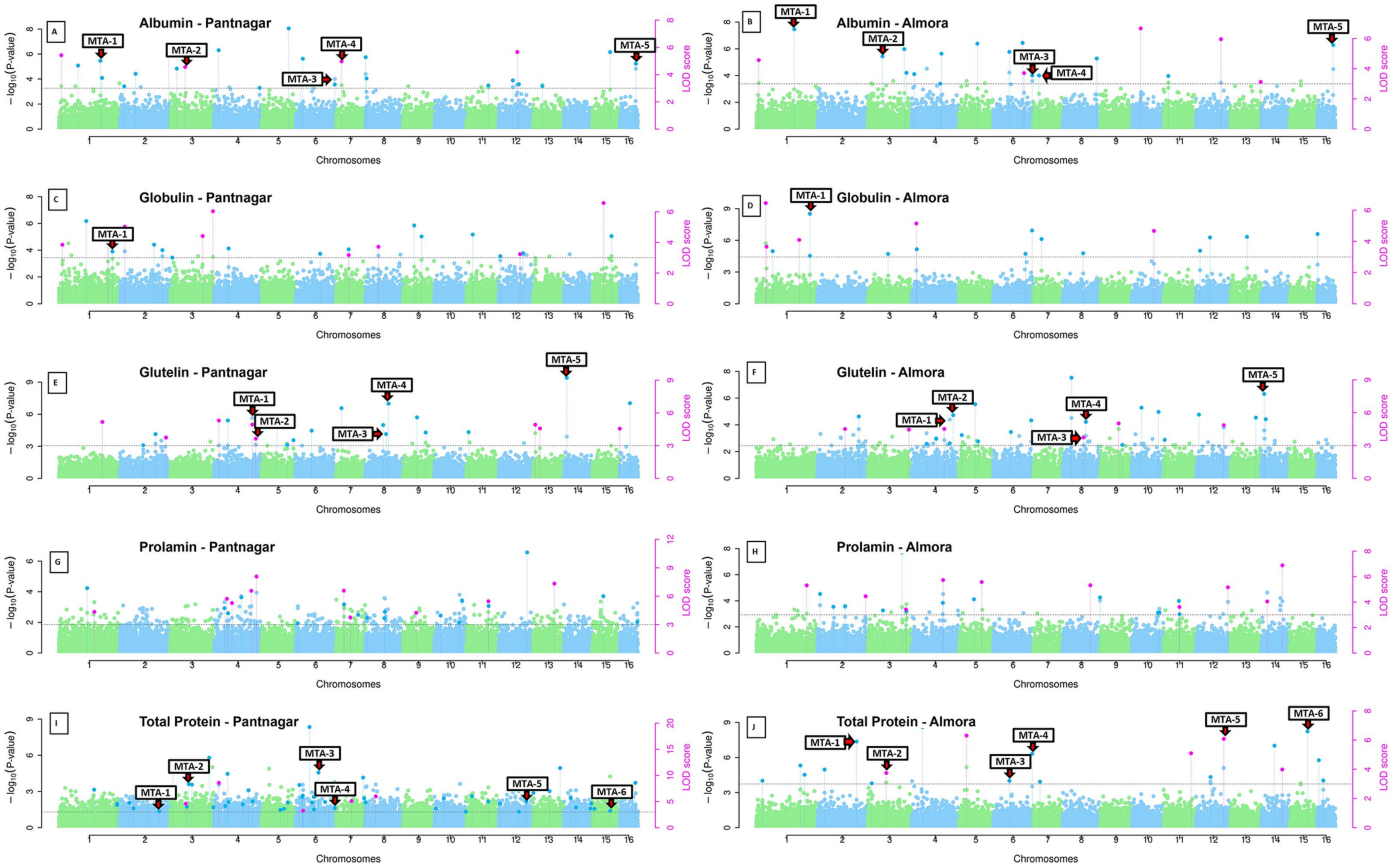
Genome-wide association mapping of accumulation of seed protein fractions in *A. hypochondriacus* across two contrasting environments. Manhattan plots showing -log₁₀(*p*-values) of marker-trait associations for albumin, globulin, glutelin, prolamin, and total protein content evaluated in Pantnagar (left panels) and Almora (right panels). The x-axis represents the 16 scaffolds (chromosomes) with alternating green and blue colors, and the y-axis shows the statistical significance of associations. Magenta points indicate LOD scores (right y-axis). Horizontal dashed lines represent the genome-wide significance threshold (LOD ≥ 3.0). Prominent peaks indicate major quantitative trait loci, with environment-specific and stable associations visible across protein fractions.

Multi-locus genome-wide association analysis was performed on 192 diverse *Amaranthus hypochondriacus* accessions evaluated across two contrasting environments for five seed protein fractions: albumin, globulin, glutelin, prolamin, and total protein (Figure 2). Population structure was rigorously controlled using five principal components derived from genome-wide SNP data. Employing five complementary multi-locus GWAS algorithms (mrMLM, FASTmrMLM, FASTmrEMMA, pKWmEB, and pLARmEB), a total of 356 significant marker-trait associations (MTAs) were identified at a stringent LOD threshold of ≥3.0, corresponding to 241 unique SNP loci distributed across the amaranth genome.

The detected MTAs exhibited substantial variation in effect sizes and trait-specific genetic architectures across the five seed protein fractions. For glutelin, 68 MTAs were identified with phenotypic variance explained ranging from 0.23 to 35.86% (mean r^2^ = 7.41%, median = 6.55%). The most significant association in the entire study was detected at Scaffold_8:4068548 for glutelin content in environment 2 (r^2^ = 35.86%, LOD = 9.19), representing an exceptionally large-effect locus for this protein fraction. Globulin exhibited 60 MTAs with r^2^ ranging from 0.70 to 23.97% (mean = 7.59%, median = 6.83%), with the major locus Scaffold_1:3707327 explaining 23.97% of phenotypic variance in environment 2 (LOD = 6.15). For albumin, 56 MTAs were identified with effect sizes ranging from 0.91 to 23.18% (mean r^2^ = 8.21%, median = 7.55%), the largest being Scaffold_16:13391891 which explained 23.18% of albumin variance in environment 2 (LOD = 5.57). Prolamin showed 77 MTAs distributed across r^2^ values from 1.76 to 18.25% (mean = 6.88%, median = 6.56%), with the top association at Scaffold_2:23995554 accounting for 18.25% of variation (LOD = 3.68). Total protein, being a composite trait integrating all fractions, exhibited the highest number of associations with 95 MTAs, though with generally smaller individual effects ranging from 0.13 to 20.14% (mean r^2^ = 5.03%, median = 4.11%), the maximum explained by Scaffold_4:2253053 in environment 1 (r^2^ = 20.14%, LOD = 8.35). Across all 356 MTAs detected for the five protein fractions, the overall distribution of effect sizes averaged 6.82% (median = 6.40%), indicating a predominantly moderate-effect genetic architecture characteristic of quantitative nutritional traits under polygenic control.

**Figure 3:**
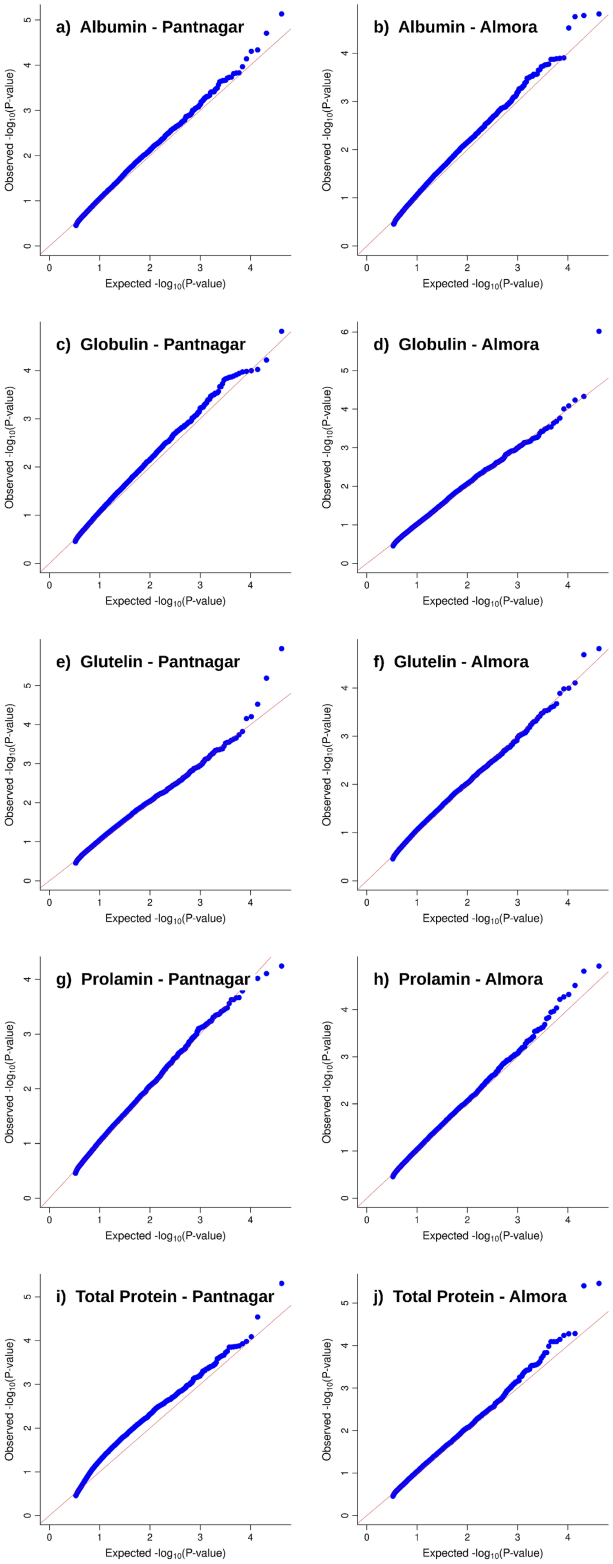
Quantile-quantile (QQ) plots for seed protein fractions across two environments. QQ plots compare expected versus observed −log₁₀(*p*-values) for marker-trait associations from the stage 1 genome-wide single-marker scan. Panels show results for five seed protein fractions in Pantnagar (left panels: **a,c,e,g,i**) and Almora (right panels: **b,d,f,h,j**) environments: **(a)** Albumin–Pantnagar, **(b)** Albumin–Almora, **(c)** Globulin–Pantnagar, **(d)** Globulin–Almora, **(e)** Prolamin–Pantnagar, **(f)** Prolamin–Almora, **(g)** Glutelin–Pantnagar, **(h)** Glutelin–Almora, **(i)** Total protein–Pantnagar, **(j)** Total protein–Almora. Points following the diagonal line indicate adherence to expected p-value distributions under the null hypothesis. Minimal deviation from the diagonal and controlled tail inflation demonstrate effective population structure correction using five principal components and EMMA kinship matrix, confirming statistical validity of detected associations. These QQ plots are identical for all six multi-locus methods applied (mrMLM, FASTmrMLM, FASTmrEMMA, pLARmEB, pKWmEB, ISIS EM-BLASSO) because they display p-values from the common stage 1 random-SNP-effect mixed linear model (RMLM) screening step (56), prior to the method-specific stage 2 multi-locus analysis with LOD ≥ 3.0 threshold.

Genome-wide association scans revealed distinct marker-trait association patterns across the five protein fractions and two environments (Figure 2). Manhattan plots demonstrated associations distributed across all 16 scaffolds, with some scaffolds exhibiting prominent peaks for specific protein fractions (e.g., Scaffold 8 for glutelin, Scaffold 16 for albumin), while others showed more dispersed signal patterns. Environment-specific peaks were evident for several protein fractions, with some associations unique to Pantnagar or Almora, alongside shared peaks detected in both environments representing the environmentally stable MTAs. Quantile-quantile plots showed good adherence to expected *p*-value distributions with controlled genomic inflation (Figure 3), indicating that population structure correction using five principal components effectively controlled for false positives while maintaining statistical power to detect true associations.

### 3.4 Environmentally stable MTAs: key features and breeding priorities

#### 3.4.1 Overview of stable associations

Applying stringent cross-environmental validation (LOD ≥ 3.0 in both Pantnagar and Almora), we identified 17 environmentally stable MTAs distributed across four protein fractions: total protein (6 MTAs), albumin (5 MTAs), glutelin (5 MTAs), and globulin (1 MTA; Table 3). Notably, no stable associations were detected for prolamin, indicating this fraction is governed by environment-specific genetic control.

**Table 3:** Environmentally stable MTAs for seed protein content in *A. hypochondriacus* and their level of confidences and contributions to trait.

Trait	SNP_ID	Avg r^2^ (%)	Avg LOD	Avg -log10(P)	Avg effect	MAF
Albumin (MTA − 1)	Scaffold_1:915141	11.59	7.37	16.99	13.99	0.197
Albumin (MTA − 2)	Scaffold_3:6633466	6.09	4.77	10.99	9	0.219
Albumin (MTA − 3)	Scaffold_6:24070042	4.51	3.41	7.86	7.64	0.174
Albumin (MTA − 4)	Scaffold_7:9227066	7.42	4.97	11.45	9.7	0.2
Albumin (MTA − 5)	Scaffold_16:13391891	15.6	5.19	11.95	24.68	0.188
Globulin (MTA − 1)	Scaffold_1:34933442	8.2	4.59	10.57	4.5	0.163
Glutelin (MTA − 1)	Scaffold_4:24796848	8.05	4.84	11.16	4.33	0.18
Glutelin (MTA − 2)	Scaffold_4:25978077	5.36	5.02	11.57	3.36	0.169
Glutelin (MTA − 3)	Scaffold_8:10163443	4.17	4.21	9.7	3.02	0.321
Glutelin (MTA − 4)	Scaffold_8:11081595	10.8	6.01	13.84	4.49	0.253
Glutelin (MTA − 5)	Scaffold_14:4246466	19.94	8.47	19.51	8.18	0.129
Total Protein (MTA − 1)	Scaffold_2:31320123	8.2	4.51	10.4	11.29	0.216
Total Protein (MTA − 2)	Scaffold_3:8414304	6.18	6.39	14.73	8.74	0.184
Total Protein (MTA − 3)	Scaffold_6:24070042	5.69	4.41	10.15	8.77	0.176
Total Protein (MTA − 4)	Scaffold_6:14594517	5.36	4.01	9.25	9.93	0.147
Total Protein (MTA − 5)	Scaffold_12:16072752	3.85	6.58	15.16	19.31	0.26
Total Protein (MTA − 6)	Scaffold_15:8325651	9.89	4.91	11.31	15.25	0.134

These stable MTAs explained 3.85 to 19.94% of phenotypic variance (mean r^2^ = 8.31%), with LOD scores ranging from 3.41 to 8.47 and minor allele frequencies from 0.129 to 0.321. All 17 stable MTAs maintained 100% directional consistency across environments, with no allele sign reversals, confirming they represent robust genetic determinants suitable for marker-assisted selection across diverse growing conditions.

#### 3.4.2 Trait-specific stable MTAs and breeding implications

Albumin (5 stable MTAs): The major albumin locus at Scaffold_16:13391891 explained 15.60% of variance (LOD = 5.19, MAF = 0.188), representing the largest single-locus effect for this fraction. The most statistically robust association at Scaffold_1:915141 achieved tri-method validation with r^2^ = 11.59% and favorable allele frequency (MAF = 0.197), making it an ideal target for immediate breeding deployment. The remaining three albumin MTAs (Scaffolds 3, 6, and 7) showed moderate but consistent effects (4.51–7.42% variance explained).

Glutelin (5 stable MTAs): Scaffold_14:4246466 harbored the most powerful stable association across all protein fractions (r^2^ = 19.94%, LOD = 8.47), though its lower MAF (0.129) may require population enrichment strategies. Two adjacent MTAs on Scaffold 4 (positions 24.8 and 26.0 Mb, separated by ~1.2 Mb) with comparable effects (r^2^ = 8.05 and 5.36%) suggest a regulatory hotspot that could be leveraged through haplotype-based selection. The two Scaffold 8 loci (positions 10.2 and 11.1 Mb) provide additional stable targets for glutelin improvement.

Total Protein (6 stable MTAs): These associations showed more moderate individual effects (3.85–9.89% variance) but offer broad applicability due to total protein’s integrative nature. The most robust locus at Scaffold_15:8325651 (r^2^ = 9.89%, MAF = 0.134) and the tri-method validated Scaffold_3:8414304 (r^2^ = 6.18%, LOD = 6.39) represent high-confidence targets. Notably, Scaffold_12:16072752 achieved quad-method validation with the highest LOD score (6.58) despite modest variance explained (3.85%), indicating exceptional statistical robustness.

Globulin (1 stable MTA): A single stable association at Scaffold_1:34933442 (r^2^ = 8.20%, LOD = 4.59, MAF = 0.163) was detected exclusively by pKWmEB, suggesting globulin content may be more environmentally sensitive than other fractions or controlled by predominantly environment-specific loci beyond this stable region.

### 3.5 Pleiotropic loci and synergistic effects

Among 9 pleiotropic MTAs affecting multiple protein fractions, 4 exhibited environmental stability for at least one trait (Table 4). Importantly, all pleiotropic effects were synergistic—favorable alleles simultaneously increased multiple protein fractions without antagonistic trade-offs, enabling cumulative improvements through allele pyramiding.

**Table 4:** Pleiotropic MTAs with environmental stability components.

SNP_ID	Protein traits	Stability pattern	Avg r^2^ (%)	Max r^2^ (%)
Scaffold_15:8325651	Albumin, Total Protein	Total Protein stable (Pantnagar+Almora), Albumin conditional (Pantnagar only)	9.15	15.57
Scaffold_6:24070042	Albumin, Total Protein	BOTH traits fully stable (Pantnagar+Almora)	5.1	10.65
Scaffold_6:14594517	Albumin, Total Protein	Total Protein stable (Pantnagar+Almora), Albumin conditional (Almora only)	6.55	9.43
Scaffold_12:16072752	Glutelin, Total Protein	Total Protein stable (Pantnagar+Almora), Glutelin conditional (Almora only)	3.23	4.69

#### 3.5.1 Fully stable pleiotropic locus

The locus at Scaffold_6:24070042 represents the ideal genetic architecture for marker-assisted selection, exhibiting complete environmental stability for both albumin and total protein across Pantnagar and Almora. This MTA was detected by three independent methods (FASTmrMLM, mrMLM, pKWmEB) and explained 4.51 and 5.69% of phenotypic variance for albumin and total protein, respectively, (average r^2^ = 5.10%, maximum r^2^ = 10.65%). The moderate favorable allele frequency (MAF = 0.174) makes this locus readily accessible for selection programs targeting simultaneous improvement of multiple protein fractions without concern for genotype-by-environment interactions.

#### 3.5.2 Hierarchically stable pleiotropic loci

Three loci demonstrated hierarchical stability patterns where total protein effects remained stable across both environments while specific protein fraction effects were environment-conditional. Scaffold_15:8325651: Stable total protein effects with albumin effects conditional on Pantnagar environment (average r^2^ = 9.15%, maximum r^2^ = 15.57%, LOD = 5.28, MAF = 0.134). Scaffold_6:14594517: Stable total protein effects with albumin effects conditional on Almora environment (average r^2^ = 6.55%, maximum r^2^ = 9.43%, LOD = 4.56, MAF = 0.147). Scaffold_12:16072752: Stable total protein effects with glutelin effects conditional on Almora environment (average r^2^ = 3.23%, maximum r^2^ = 4.69%, LOD = 5.31, MAF = 0.266).

This hierarchical pattern suggests dual functional components: a constitutive module controlling overall protein accumulation and environment-responsive elements modulating specific fraction biosynthesis. Such loci offer robust total protein improvement while requiring environmental consideration for fraction-specific manipulation.

### 3.6 Genomic distribution and clustering patterns

#### 3.6.1 Genome-wide distribution

The 17 environmentally stable MTAs were distributed across 12 distinct scaffolds, with uneven distribution indicating the presence of regulatory hotspots (Table 5). Three scaffolds (6, 8, and 4) harbored multiple stable MTAs, while nine scaffolds contained single associations.

**Table 5:** Scaffold distribution of environmentally stable MTAs.

Scaffold	No. MTAs	Protein traits	Physical span
Scaffold 6	3	Albumin (1), Total Protein (2)	~19 Mb region (5.0–24.1 Mb)
Scaffold 8	2	Glutelin (2)	Positions 10.2 Mb and 11.1 Mb
Scaffold 4	2	Glutelin (2)	Adjacent at 24.8–26.0 Mb (~1.2 Mb separation)
Scaffold 1	2	Albumin (1), Globulin (1)	Distinct loci at 0.9 Mb and 34.9 Mb
Scaffold 3	2	Albumin (1), Total Protein (1)	Independent loci at 6.6 Mb and 8.4 Mb
Scaffolds 2, 7, 12, 14, 15, 16	1 each	Various	Single stable MTAs

#### 3.6.2 Notable genomic hotspots

Three genomic regions emerged as regulatory hotspots warranting detailed characterization:

Scaffold 6 (albumin-total protein hub): This scaffold contained three stable MTAs spanning ~19 Mb, with two pleiotropic loci at positions 14.6 and 24.0 Mb (separated by ~9.5 Mb) both affecting albumin and total protein. The latter locus represents the fully stable pleiotropic MTA described in Section 3.6.1. This clustering suggests Scaffold 6 harbors coordinated regulatory networks controlling albumin biosynthesis and its contribution to total protein content, possibly through linked genes in protein synthesis or trafficking pathways.

Scaffold 8 (glutelin regulation center): Two stable glutelin MTAs at positions 10.2 and 11.1 Mb (separated by ~0.9 Mb) likely represent components of a glutelin regulatory module rather than independent loci. Both show comparable effect sizes and consistent environmental stability. Additionally, this scaffold harbored the largest-effect environment-specific MTA at position 4.1 Mb (r^2^ = 35.86% for glutelin in Almora), suggesting Scaffold 8 contains both stable and environment-responsive glutelin regulation mechanisms that could be leveraged for breeding in specific target environments.

Scaffold 4 (linked glutelin loci): Two stable glutelin MTAs separated by ~1.2 Mb (positions 24.8 and 26.0 Mb) with similar effect sizes (r^2^ = 8.05 and 5.36%) and allele frequencies (MAF = 0.18 and 0.17) present an intriguing architecture. These could represent: (1) independent genes in linkage disequilibrium, (2) a single gene with multiple functional polymorphisms, or (3) closely linked pathway components. Fine-mapping this region would inform optimal marker selection strategies—if independent, both loci could be pyramided for additive effects; if representing a single gene, haplotype-based selection would be more appropriate.

### 3.7 Candidate gene search and identification

We analyzed genes within 250 kb windows (based on LD decay) flanking each significant SNP and identified 48 candidate genes based on functional annotations (Table 6). The candidates were distributed as follows: 12 genes at albumin loci, 5 genes at the globulin locus, 15 genes at glutelin loci, and 18 genes at total protein loci (with overlap at the pleiotropic locus).

**Table 6:** The identified candidate genes and the functional category and the rationale for selection.

Trait	MTA	Gene_ID	Gene name	Functional category
Albumin	MTA1	AH000091	B3-domain transcription factor (VRN1-like)	Transcriptional regulation
Albumin	MTA1	AH000077	ERGIC3 (ER-Golgi intermediate compartment protein)	Protein trafficking
Albumin	MTA1	AH000099	Arogenate dehydratase 2 (ADT2)	Amino acid biosynthesis
Albumin	MTA1	AH000113	Asparagine-tRNA ligase (SYNC1)	Translation machinery
Albumin	MTA2	AH005330	Serine hydroxymethyltransferase 6 (SHMT6)	Amino acid metabolism
Albumin	MTA2	AH005322	ATP phosphoribosyltransferase (histidine biosynthesis)	Amino acid biosynthesis
Albumin	MTA3	AH011207	VAMP726 (vesicle-associated membrane protein)	Protein trafficking
Albumin	MTA3	AH011199	LEA protein (Late embryogenesis abundant)	Seed development
Albumin	MTA4	AH011500	IKU1 (HAIKU1)	Seed development
Albumin	MTA4	AH011491	LEA6 (Late embryogenesis abundant protein 6)	Seed development
Albumin	MTA4	AH011486	NPF7.3/NRT1.5 (nitrate transporter)	Nitrogen transport
Albumin	MTA5	AH023554	Basic pentacysteine 6 (BPC6)	Transcriptional regulation
Globulin	MTA1	AH002203	KDEL receptor (ER lumen protein-retaining receptor)	ER quality control
Globulin	MTA1	AH002173	DnaJ/Hsp40 chaperone	Protein folding
Globulin	MTA1	AH002196	ERGIC3 (ER-Golgi intermediate compartment protein)	Protein trafficking
Globulin	MTA1	AH002195	Glutamate decarboxylase (GAD)	Amino acid metabolism
Globulin	MTA1	AH002201	GASA1 (Gibberellin-regulated protein 1)	Hormone signaling
Glutelin	MTA1	AH007945	NCED3 (9-cis-epoxycarotenoid dioxygenase)	Hormone biosynthesis
Glutelin	MTA1	AH007946	NCED1 (9-cis-epoxycarotenoid dioxygenase)	Hormone biosynthesis
Glutelin	MTA1	AH007906	Cyclophilin 38 (peptidyl-prolyl isomerase)	Protein folding
Glutelin	MTA1	AH007909	NAC078/NAC050 transcription factor	Transcriptional regulation
Glutelin	MTA2	AH008026	bZIP61 transcription factor	Transcriptional regulation
Glutelin	MTA2	AH008027	bZIP61 transcription factor (copy 2)	Transcriptional regulation
Glutelin	MTA2	AH008045	CUC1/NAC054 (NAC transcription factor)	Transcriptional regulation
Glutelin	MTA2	AH008046	CUC1/NAC054 (NAC transcription factor, copy 2)	Transcriptional regulation
Glutelin	MTA2	AH008041	NUP88 (Nuclear pore complex protein)	mRNA export
Glutelin	MTA3	AH013418	ABF2/AREB1 (ABA-responsive element binding factor)	Transcriptional regulation
Glutelin	MTA3	AH013394	NUP107 (Nuclear pore complex protein)	mRNA export
Glutelin	MTA3	AH013407	HSP17.4B (Small heat shock protein)	Protein folding/Stress response
Glutelin	MTA4	AH013498	Delta VPE (Vacuolar processing enzyme)	Protein processing
Glutelin	MTA4	AH013474	Alpha-mannosidase 2 (MNS2)	Glycan processing (ER/Golgi)
Glutelin	MTA5	AH020723	ATS3A (Embryo-specific protein 3)	Seed development
TotalProtein	MTA1	AH004248	CCT1/TCP-1 (T-complex protein 1 alpha subunit)	Protein folding
TotalProtein	MTA1	AH004225	ARF1 (Auxin response factor 1)	Transcriptional regulation
TotalProtein	MTA1	AH004233	ALFIN-like 6 (PHD finger protein)	Chromatin regulation
TotalProtein	MTA1	AH004234	ALFIN-like 7 (PHD finger protein)	Chromatin regulation
TotalProtein	MTA1	AH004235	ALFIN-like 7 (PHD finger protein, copy 2)	Chromatin regulation
TotalProtein	MTA1	AH004236	NAC035 (NAC domain transcription factor)	Transcriptional regulation
TotalProtein	MTA2	AH005464	Glutamine-tRNA ligase (Glutaminyl-tRNA synthetase)	Translation machinery
TotalProtein	MTA2	AH005467	Cysteine-tRNA ligase (Cysteinyl-tRNA synthetase)	Translation machinery
TotalProtein	MTA2	AH005486	BAT1 (Bidirectional amino acid transporter)	Amino acid transport
TotalProtein	MTA2	AH005461	bZIP43 (Basic leucine zipper transcription factor)	Transcriptional regulation
TotalProtein	MTA2	AH005474	AATL1 (Lysine/histidine transporter)	Amino acid transport
TotalProtein	MTA3	AH010392	PIR transcription activator (SRA1)	Transcriptional regulation
TotalProtein	MTA4	[Shared with Albumin MTA3]	[See Albumin candidates above]	[Multiple pathways]
TotalProtein	MTA5	AH019095	3-ketoacyl-ACP synthase III	Fatty acid biosynthesis
TotalProtein	MTA5	AH019093	L-aspartate oxidase (AO)	Amino acid metabolism
TotalProtein	MTA5	AH019092	ATX1 (Trithorax/Histone methyltransferase)	Chromatin regulation
TotalProtein	MTA6	AH022443	WRKY34 transcription factor	Transcriptional regulation

Functional categories of the 39 candidates included: transcriptional regulators (13 genes, 26%), amino acid metabolism and transport genes (7 genes, 14%), protein folding and quality control genes (4 genes, 8%), seed development genes (4 genes, 8%), chromatin regulators (4 genes, 8%), protein trafficking genes (3 genes, 6%), translation-related genes (3 genes, 6%), hormone biosynthesis and signaling genes (3 genes, 6%), mRNA export genes (2 genes, 4%), and other categories (7 genes, 14%). No genes encoding 11S globulins, 7S globulins, 2S albumins, or glutelins were identified within the 250 kb windows.

#### 3.7.1 Glutelin-associated loci

The five glutelin MTAs contained 15 candidate genes. At Scaffold_4:24,796,848, two tandemly duplicated genes (AH007945 and AH007946) encoded 9-*cis*-epoxycarotenoid dioxygenase (NCED). This locus also contained AH007906 (cyclophilin 38) and AH007909 (NAC078 transcription factor). At Scaffold_4:25,978,077, two tandemly duplicated genes (AH008026 and AH008027) encoded bZIP61 transcription factors. This locus also harbored two NAC domain transcription factor genes (AH008045 and AH008046) and AH008041 encoding nuclear pore complex protein NUP88. The MTA at Scaffold_8:10,163,443 contained AH013418 encoding ABF2/AREB1 (a bZIP transcription factor), AH013394 encoding nuclear pore complex protein NUP107, and AH013407 encoding a small heat shock protein (HSP17.4B). At Scaffold_8:11,081,595, candidates included AH013498 encoding delta-type vacuolar processing enzyme (VPE) and AH013474 encoding alpha-mannosidase 2. The Scaffold_14:4,246,466 MTA contained AH020723 encoding embryo-specific protein ATS3A.

#### 3.7.2 Globulin-associated locus

The single globulin MTA on Scaffold_1:34,933,442 contained five candidate genes. These included AH002203 encoding a KDEL receptor, AH002173 encoding a DnaJ/Hsp40 chaperone, AH002196 encoding ERGIC3 (ER-Golgi intermediate compartment protein), AH002195 encoding glutamate decarboxylase, and AH002201 encoding a GASA1 gibberellin-regulated protein.

#### 3.7.3 Albumin-associated loci

The five albumin MTAs contained 12 candidate genes. At Scaffold_1:915,141, candidates included AH000091 (B3 domain-containing transcription factor), AH000077 (ERGIC3), AH000113 (asparagine-tRNA ligase), and AH000099 (arogenate dehydratase 2). At Scaffold_3:6,633,466, candidates included AH005330 (serine hydroxymethyltransferase 6) and AH005322 (ATP phosphoribosyltransferase for histidine biosynthesis). The Scaffold_6:24,070,042 locus (shared with total protein) contained AH011207 (VAMP726), AH011199 (late embryogenesis abundant protein), and multiple wall-associated receptor kinase genes. At Scaffold_7:9,227,066, candidates included AH011500 (IKU1/HAIKU1), AH011491 (LEA6 protein), and AH011486 (NPF7.3 nitrate transporter). The Scaffold_16:13,391,891 MTA contained AH023554 (basic pentacysteine 6 transcription factor).

#### 3.7.4 Total protein-associated loci

The six total protein MTAs contained 18 candidate genes. At Scaffold_2:31,320,123, candidates included three tandemly duplicated ALFIN-like PHD finger proteins (AH004233, AH004234, AH004235), AH004225 (ARF1 auxin response factor), AH004236 (NAC035 transcription factor), and AH004248 (CCT1 chaperonin subunit). At Scaffold_3:8,414,304, candidates included AH005464 (glutamine-tRNA synthetase), AH005467 (cysteine-tRNA synthetase), AH005486 (BAT1 amino acid transporter), AH005461 (bZIP43 transcription factor), and AH005474 (lysine/histidine amino acid transporter). At Scaffold_6:14,594,517, the candidate was AH010392 (PIR transcription activator). The Scaffold_6:24,070,042 locus shared with albumin is described above. At Scaffold_12:16,072,752, candidates included AH019095 (3-ketoacyl-ACP synthase III), AH019093 (L-aspartate oxidase), and AH019092 (ATX1 histone methyltransferase). At Scaffold_15:8,325,651, candidates included AH022443 (WRKY34 transcription factor).

#### 3.7.5 Tandem gene duplications at significant loci

Tandem gene duplications were observed at multiple MTAs. The glutelin locus at Scaffold_4:24,796,848 contained two NCED genes. The glutelin locus at Scaffold_4:25,978,077 contained two bZIP61 genes and two CUC1/NAC transcription factor genes. The total protein locus at Scaffold_2:31,320,123 contained three ALFIN-like genes.

#### 3.7.6 Overlapping gene families across traits

The ERGIC3 gene family appeared at two loci: AH000077 at the albumin locus (Scaffold_1:915,141) and AH002196 at the globulin locus (Scaffold_1:34,933,442), separated by approximately 34 Mb. Nuclear pore complex proteins appeared at two glutelin loci: NUP88 (AH008041) at Scaffold_4:25,978,077 and NUP107 (AH013394) at Scaffold_8:10,163,443. bZIP transcription factors were found at glutelin loci (AH013418, AH008026, AH008027) and a total protein locus (AH005461). Late embryogenesis abundant (LEA) proteins appeared at two albumin loci: AH011491 at Scaffold_7:9,227,066 and AH011199 at Scaffold_6:24,070,042.

### 3.8 Functional enrichment analysis of candidate genes

To gain insights into the biological functions and molecular mechanisms underlying the identified candidate genes, Gene Ontology (GO) enrichment and Kyoto Encyclopedia of Genes and Genomes (KEGG) pathway analyses were performed on the 48 candidate genes identified from trans-regulatory loci associated with seed protein fractions.

Of the 48 candidate genes, 30 (62.5%) possessed GO term annotations and 17 (35.4%) had KEGG Orthology (KO) identifiers based on the *A. hypochondriacus* v2.1 genome annotation. Given the modest candidate gene set size and incomplete functional annotation coverage in the non-model organism amaranth, we adopted a false discovery rate (FDR) threshold of 0.10 for identifying significantly enriched terms. This relaxed threshold is appropriate for small gene sets from non-model organisms where statistical power is inherently limited due to annotation gaps and sample size constraints, and has been recommended for hypothesis generation in functional genomics studies. The enrichment analysis employed a hypergeometric test with Benjamini-Hochberg FDR correction, using the entire annotated genome as the background reference.

GO enrichment analysis revealed 13 significantly enriched terms (FDR < 0.10) across molecular function and cellular component categories. Among molecular function terms, the most significant enrichment was observed for “histone binding” (GO:0042393; FDR = 0.035), with two candidate genes (AH004234 and AH004235) encoding Alfin-like 7 (AL7) proteins containing PHD finger domains that recognize methylated histone H3K4 marks. Additional molecular function enrichments included “aminoacyl-tRNA ligase activity” (GO:0004812; FDR = 0.057) for genes AH000113 and AH005464, “sequence-specific DNA binding” (GO:0043565; FDR = 0.057) for three transcription factors, and “DNA-binding transcription factor activity” (GO:0003700; FDR = 0.084).

Notably, enrichment of “ER retention sequence binding” (GO:0046923; FDR = 0.084) was detected for gene AH002203, encoding a KDEL receptor responsible for retrieving endoplasmic reticulum (ER)-resident chaperones from the Golgi apparatus. This finding provides statistical support for ER protein quality control mechanisms limiting storage protein accumulation. Additional enriched terms included “amino acid transmembrane transporter activity” (GO:0015171; FDR = 0.084) for an amino acid transporter (AH005486), and metabolic enzyme activities including “mannosyl-oligosaccharide 1,2-alpha-mannosidase activity” (GO:0004571; FDR = 0.057) and “prephenate dehydratase activity” (GO:0004664; FDR = 0.057). Cellular component enrichments included “nuclear pore” (GO:0005643; FDR = 0.076) and “cytoplasm” (GO:0005737; FDR = 0.076).

KEGG pathway analysis identified significant enrichment of the aminoacyl-tRNA biosynthesis pathway (map00970; FDR = 0.020). Three candidate genes were mapped to this pathway: AH000113 encoding asparaginyl-tRNA synthetase, AH005464 encoding glutaminyl-tRNA synthetase, and AH005467 encoding cysteinyl-tRNA synthetase. These aminoacyl-tRNA synthetases catalyze the attachment of specific amino acids to their cognate tRNAs, representing the first committed step in protein translation. Notably, AH000113 was associated with albumin content, while AH005464 and AH005467 were associated with total protein content, indicating that genetic variation in translation machinery components may directly impact seed protein accumulation capacity. The consistency between GO-level enrichment of aminoacyl-tRNA ligase activity and KEGG pathway enrichment of aminoacyl-tRNA biosynthesis provides robust statistical validation for translation machinery as a bottleneck in seed protein accumulation. Complete enrichment statistics including *p*-values, FDR values, gene ratios, and gene identifiers are provided in Supplementary material 5 (GO enrichment) and Supplementary material 6 (KEGG pathway enrichment).

The functional enrichment results provide statistical support for the hypothesis that trans-regulatory loci control seed protein content through coordinated regulation at multiple levels: (1) translation capacity limitation via aminoacyl-tRNA synthetases, (2) ER protein quality control via KDEL receptor-mediated chaperone retrieval, (3) transcriptional regulation via DNA-binding transcription factors, and (4) chromatin-level regulation via histone-binding proteins. Importantly, no storage protein structural genes were identified among the candidate genes, reinforcing the predominance of regulatory variation in controlling seed protein composition.

## 4 Discussion

### 4.1 Phenotypic variation, heritability, and genetic architecture of seed protein fractions

The evaluation of 192 grain amaranth accessions across contrasting environments at Pantnagar and Almora revealed substantial phenotypic variation in seed protein fraction composition, with all five traits displaying continuous variation characteristic of quantitative traits under polygenic control. Amaranth’s high genetic variability and adaptability to diverse environmental conditions have been well-documented, allowing this pseudocereal to thrive under various climatic scenarios from tropical to temperate regions (23). The environmental conditions at Almora significantly enhanced protein accumulation, with total protein content increasing by 24% compared to Pantnagar (231.19 g/kg vs. 186.59 g/kg), and albumin showing the most dramatic response with a 39% increase. These differential environmental responses suggest that protein fractions are subject to distinct regulatory mechanisms with differing environmental sensitivities, underscoring the necessity of multi-environment trials for effective selection.

The genetic parameter analysis reveals distinct patterns of inheritance and selection potential across seed protein fractions. The high heritabilities observed for albumin (H^2^ = 0.933), total protein (H^2^ = 0.910), and glutelin (H^2^ = 0.873) indicate that phenotypic variation for these traits is predominantly determined by genetic factors, with minimal environmental influence. These high heritabilities suggest that selection for these fractions will be highly effective, with phenotypic selection closely reflecting genotypic value. Such heritability estimates are consistent with or exceed those reported for protein content in other grain crops, where seed protein concentration typically exhibits moderate to high heritability ranging from 0.43 to 0.85 depending on population structure and environmental conditions (24–26). High heritability coupled with high genetic advance, as observed for albumin, indicates the predominance of additive gene action and suggests that selection would be highly effective for trait improvement (24, 27). In contrast, the moderate heritability of prolamin (H^2^ = 0.523) indicates substantial environmental influence on this fraction, requiring more intensive phenotyping across multiple environments or genomic selection approaches to achieve equivalent genetic gains (28, 29).

Genetic advance as percentage of mean (GA%) provides a unified metric for comparing selection potential across traits with different scales. Albumin’s exceptionally high GA% (60.78%) indicates that one cycle of selection at 5% intensity would produce a population mean shift of approximately 61% of the current mean, representing remarkable improvement potential that combines high heritability with substantial genetic variance. Globulin, glutelin, and total protein show moderate but favorable GA% values (26–28%), indicating effective selection response. Traits exhibiting high heritability and moderate to high genetic advance as percentage of mean are particularly amenable to improvement through phenotypic selection schemes (30). The identification of 17 environmentally stable marker-trait associations through multi-locus GWAS, which detected moderate-effect loci that would be missed by traditional single-locus approaches (31), provides immediate tools for marker-assisted selection. These stable markers, combined with high heritability estimates, enable breeders to select superior genotypes without extensive phenotyping across multiple environments (32).

### 4.2 Biochemical nature of Osborne fractions and implications for genetic analysis

Our study employed Osborne fractionation to characterize seed protein composition in amaranth. It is important to clarify that while these fractions are often referred to in the literature as “storage protein fractions,” they represent biochemical classifications based on differential solubility rather than pure functional categories. The albumin fraction contains water-soluble proteins including storage albumins but also metabolic enzymes, ribosomal proteins, and other cytoplasmic proteins. Similarly, the globulin fraction, while enriched in 7S and 11S storage globulins, contains diverse salt-soluble proteins. The glutelin fraction represents alkali-soluble proteins including glutelin-type storage proteins, and the prolamin fraction contains alcohol-soluble proteins which are minimal in amaranth compared to cereals.

Despite this biochemical heterogeneity, our quantitative analysis of these fractions remains biologically meaningful for several reasons. First, in mature amaranth seeds, storage proteins constitute the vast majority (>70%) of total seed protein, with the Osborne fractions dominated by proteins serving nitrogen reserve functions (6, 7). Second, from a breeding perspective, the traits we measured—total protein content per fraction—represent the phenotypes of direct interest for nutritional biofortification, regardless of whether each fraction contains exclusively storage proteins or a mixture enriched in storage proteins. Third, our genetic analysis targeted natural variation in the overall protein accumulation machinery and nitrogen allocation to developing seeds, which affects both storage proteins and their associated biosynthetic and regulatory proteins coordinately.

The trans-regulatory architecture we identified—with no structural storage protein genes among our significant MTAs—actually reinforces that we are capturing variation in the fundamental cellular processes governing seed protein accumulation: transcriptional regulation, hormone signaling, amino acid metabolism and transport, protein synthesis capacity, and protein folding/trafficking machinery. These upstream regulatory networks control the coordinated accumulation of all seed proteins, with storage proteins being the quantitatively dominant beneficiaries given their abundance during seed filling.

### 4.3 Trans-regulatory control: a paradigm shift in understanding amaranth protein variation

A striking finding of this study is the complete absence of structural seed storage protein genes within any of the 17 environmentally stable marker-trait associations. No genes encoding 11S globulins, 7S globulins, 2S albumins, or glutelins were identified within the 250 kb windows flanking significant SNPs, based on the average linkage disequilibrium decay distance for this diversity panel. This observation indicates that natural variation in amaranth seed protein content is controlled entirely through trans-regulatory mechanisms rather than through cis-regulatory or structural variation in the SSP genes themselves. This pattern is consistent with findings in other crop species where quantitative trait loci for grain protein content frequently map to regulatory loci rather than to the structural SSP genes. In rice, multiple QTL studies have identified regulatory regions controlling glutelin, prolamin, globulin, and albumin content, with amino acid transporters such as OsAAP6 shown to regulate synthesis and accumulation of multiple SSP fractions simultaneously (33). Similar patterns have been observed in wheat, where conserved cis-regulatory modules control the transcription of high-molecular-weight glutenin subunit genes through complex networks of transcription factors rather than through structural gene variation (34).

The predominance of trans-regulatory variation likely reflects strong evolutionary conservation of the structural SSP genes themselves, with natural and domestication-related selection acting instead on the regulatory networks that control their expression, processing, and accumulation. This distinction between amaranth as a dicot and cereals as monocots is particularly relevant, as amaranth utilizes B3-domain transcription factors from the LAFL network for seed maturation control, whereas cereals employ the Opaque2-Prolamin-box binding factor network for prolamin gene regulation (35). The trans-regulatory architecture identified in this study has important implications for breeding strategies, as marker-assisted selection targeting favorable alleles at these regulatory loci can enhance protein content without compromising seed protein quality or amino acid composition.

### 4.4 Multi-level regulatory control: insights from functional enrichment

Functional enrichment analysis of the 48 candidate genes provided statistical validation for multi-level regulatory control of seed protein accumulation. The most significantly enriched molecular function was “histone binding” (GO:0042393; FDR = 0.035), represented by ALFIN-like 7 proteins containing PHD finger domains that recognize methylated histone H3K4 marks. This enrichment indicates that chromatin-level epigenetic regulation represents a major control point for seed protein content variation. At the total protein locus on Scaffold_2:31,320,123, three tandemly duplicated ALFIN-like PHD finger proteins were identified alongside ATX1, a histone H3K4 methyltransferase involved in transcriptional activation through chromatin remodeling. These epigenetic regulators likely modulate the accessibility of seed maturation genes and storage protein loci to transcriptional machinery, providing a mechanism for coordinated regulation of multiple protein biosynthesis pathways.

The only significantly enriched KEGG pathway was “aminoacyl-tRNA biosynthesis” (map00970; FDR = 0.020), comprising three aminoacyl-tRNA synthetases: asparaginyl-tRNA synthetase associated with albumin content, and glutaminyl-tRNA and cysteinyl-tRNA synthetases associated with total protein content. These enzymes catalyze the attachment of specific amino acids to their cognate tRNAs, representing the first committed step in protein translation (36). The enrichment of this pathway indicates that translation capacity represents a rate-limiting bottleneck for seed protein accumulation. At the total protein locus on Scaffold_3:8,414,304, these tRNA synthetases co-localized with BAT1, a bidirectional amino acid transporter that mediates amino acid exchange across cellular membranes. This clustering suggests that both the supply of charged tRNAs for translation and the availability of free amino acids for protein synthesis jointly limit overall protein accumulation capacity.

Additionally, enrichment of “ER retention sequence binding” (GO:0046923; FDR = 0.084), represented by the KDEL receptor at the globulin locus, provides statistical support for endoplasmic reticulum protein quality control as a limiting factor. The convergence of translation machinery limitation, amino acid supply constraints, ER folding capacity, and chromatin-level regulation demonstrates that seed protein content is controlled through coordinated bottlenecks at multiple steps in the gene expression and protein biosynthesis pathway.

### 4.5 ABA signaling pathway as master regulator of glutelin accumulation

The most compelling evidence for pathway-level control of seed protein content comes from glutelin-associated loci, where multiple components of the abscisic acid signaling cascade were identified across independent genomic regions. Two tandemly duplicated NCED genes (9-cis-epoxycarotenoid dioxygenase) were identified at the glutelin locus on Scaffold_4:24,796,848. NCED catalyzes the rate-limiting step in ABA biosynthesis by cleaving 9-cis-epoxycarotenoids to produce xanthoxin, the immediate precursor of ABA (37). In developing seeds, NCED6 and NCED9 are the major genes responsible for ABA production, with endosperm-localized NCED expression being particularly critical for seed maturation processes (38, 39). Overexpression of NCED genes has been shown to increase ABA levels more than 20-fold and enhance both seed maturation and stress tolerance (40).

At an independent glutelin locus on Scaffold_8:10,163,443, we identified ABF2/AREB1, encoding a bZIP transcription factor that functions as a master regulator of ABA-responsive gene expression. ABF2/AREB1 belongs to the AREB/ABF subfamily of bZIP transcription factors that bind to ABA-responsive elements (ABREs) in target gene promoters and activate transcription of seed maturation genes (41). ABF2/AREB1 requires phosphorylation by SnRK2 protein kinases for full activation and has been shown to directly activate late embryogenesis abundant (LEA) class genes and other ABA-inducible regulatory genes, with mutant studies demonstrating dramatically reduced expression of stress-responsive and seed maturation genes (42, 43). The co-localization of NCED genes at one locus and ABF2/AREB1 at another, both associated with glutelin content, provides strong genetic evidence that the ABA biosynthesis-signaling pathway is a major determinant of glutelin accumulation in amaranth seeds. This pathway likely operates through ABA-mediated activation of seed maturation transcription factors that directly or indirectly regulate glutelin gene expression.

Additional support for ABA pathway involvement comes from the identification of two tandemly duplicated bZIP61 transcription factors at a third glutelin locus on Scaffold_4:25,978,077. While less extensively characterized than ABF2/AREB1, bZIP61 belongs to the same group-A bZIP family known to participate in ABA signaling and seed maturation processes. The presence of tandem duplications of both NCED genes and bZIP genes suggests that gene dosage effects may be important for modulating ABA pathway flux and downstream transcriptional responses during seed filling. This locus also harbored two NAC domain transcription factor genes, which are key regulators of seed development often functioning downstream of ABA signaling, and nuclear pore complex protein NUP88, potentially controlling the nucleocytoplasmic trafficking of ABA-responsive transcription factors or their target mRNAs.

### 4.6 Endoplasmic reticulum protein folding capacity limits globulin accumulation

The single environmentally stable globulin-associated locus on Scaffold_1:34,933,442 contained two key components of the endoplasmic reticulum protein quality control system: a KDEL receptor and a DnaJ/Hsp40 chaperone. This finding suggests that ER protein folding capacity represents a major bottleneck limiting globulin accumulation in amaranth seeds. The KDEL receptor is a seven-transmembrane-domain protein responsible for retrieving ER-resident chaperones from the Golgi complex back to the ER lumen (44). Major chaperones bearing KDEL-family retrieval signals include BiP (binding immunoglobulin protein), protein disulfide isomerase (PDI), and calnexin, all of which are present at millimolar concentrations in the ER and are essential for proper protein folding (45). Loss of KDEL receptor function leads to secretion of these chaperones from the cell and causes severe defects in protein folding and cellular proteostasis (46). During seed development, the massive synthesis of storage proteins places extraordinary demands on the ER folding machinery, and KDEL receptor activity is critical for maintaining adequate chaperone levels to handle this protein folding load.

The co-localization of a DnaJ/Hsp40 chaperone at the same locus further supports the interpretation that ER protein quality control capacity limits globulin accumulation. DnaJ/Hsp40 proteins function as co-chaperones of Hsp70 family members (including BiP in the ER) by binding unfolded polypeptides, stimulating Hsp70 ATPase activity, and stabilizing Hsp70-substrate interactions (47). The J-domain of DnaJ proteins is essential for activating Hsp70 by binding to its ATPase domain, while the substrate-binding domains can directly recognize exposed hydrophobic regions of misfolded proteins (48). In the ER, DnaJ/Hsp40 proteins such as ERdj3 serve as cofactors for BiP’s interactions with unfolded substrates, including newly synthesized secretory and storage proteins (49). The identification of both KDEL receptor and DnaJ/Hsp40 at a single major globulin locus suggests that natural variation in ER chaperone homeostasis and protein folding capacity is a key determinant of globulin content. This interpretation is consistent with the observation that globulin proteins, which typically form large oligomeric structures, have particularly stringent folding requirements and are especially sensitive to chaperone availability.

### 4.7 Environmentally stable genetic architecture and breeding implications

The identification of 17 environmentally stable marker-trait associations reveals robust genetic determinants of seed protein content that persist across environmental variation. The perfect directional consistency (100%) of allelic effects across environments provides strong evidence that these associations represent true functional polymorphisms rather than statistical artifacts (50). The distribution of stable MTAs across protein fractions—with total protein, albumin, and glutelin each showing multiple stable loci but globulin showing only one—indicates differential levels of environmental stability in genetic control mechanisms. The absence of environmentally stable MTAs for prolamin suggests this fraction is under strong genotype-by-environment interaction, with predominantly environment-specific genetic control (51). This finding has important breeding implications: prolamin improvement will require environment-specific marker deployment, whereas the stable MTAs for other fractions can be deployed across diverse environments.

Nine pleiotropic MTAs affecting multiple protein fractions were identified, with all pleiotropic effects being synergistic—favorable alleles simultaneously increased multiple protein fractions without antagonistic trade-offs (52). This universal synergistic pleiotropy enables cumulative improvements through allele pyramiding, as selection for favorable alleles at multiple loci will improve overall protein composition without compromising individual fractions (53). The fully stable pleiotropic locus at Scaffold_6:24,070,042, which affects both albumin and total protein across both environments with moderate allele frequency (MAF = 0.174), represents an ideal target for marker-assisted selection programs targeting simultaneous improvement of multiple protein fractions.

Several high-priority breeding targets emerged from this study. The albumin MTA at Scaffold_16:13,391,891 explained 15.60% of phenotypic variance with robust detection across both environments and moderate minor allele frequency (MAF = 0.188), making this locus readily accessible for marker-assisted selection. The glutelin MTA at Scaffold_14:4,246,466 displayed exceptional effect size (19.94%) with consistent detection, representing the most powerful stable association identified. For total protein improvement, the MTA at Scaffold_2:31,320,123 offers robust environmental stability with moderate effect size and accessible allele frequency. The genomic clustering of multiple MTAs on Scaffolds 4, 6, and 8 suggests the presence of regulatory hotspots or linked gene complexes controlling protein content, with potential for haplotype-based selection strategies to capture multiple favorable alleles simultaneously.

The trans-regulatory nature of all identified QTL has important implications for breeding strategies. Because the variants identified act through regulatory mechanisms rather than through changes in the structural SSP genes themselves, marker-assisted selection targeting favorable alleles at these regulatory loci can develop cultivars with enhanced protein content without compromising seed protein quality or amino acid composition. The co-localization of multiple pathway components at single loci—such as the NCED-ABF2 ABA pathway for glutelin, the KDEL receptor-DnaJ chaperone system for globulin, and the tRNA synthetase-amino acid transporter cluster for total protein—suggests that these loci may have particularly large effects and could be prioritized for marker-assisted selection efforts. Pyramiding favorable alleles across multiple loci targeting different regulatory bottlenecks (chromatin accessibility, ABA signaling, ER folding capacity, translation machinery) could lead to cumulative improvements in protein content through additive or synergistic effects (29, 32).

### 4.8 Comparative genomics and future directions

The trans-regulatory control of storage protein content observed in amaranth parallels findings in other crop species, though the specific genes and pathways involved show both conserved and divergent features. In rice, extensive QTL mapping studies have identified numerous loci controlling total grain protein content as well as individual SSP fractions, with many QTL co-localizing for different fractions and often mapping to regulatory genes rather than to the glutelin or prolamin structural genes themselves (33, 54). These cross-species comparisons suggest that trans-regulatory control through modulation of common pathways—including transcription factor networks, hormone signaling (particularly ABA), amino acid metabolism and transport, and protein folding/trafficking machinery—represents a widespread mechanism for generating natural variation in seed protein content across diverse crop species.

However, amaranth also shows distinctive features reflecting its phylogenetic position as a dicot with negligible prolamin content, in contrast to cereals which accumulate large amounts of prolamins. The presence of B3-domain transcription factors (LAFL network members) among amaranth seed protein fractions QTL reflects dicot-specific seed regulatory pathways that differ from the Opaque2-Prolamin-box binding factor network controlling cereal prolamin expression. The predominance of glutelin and globulin as major SSP fractions in amaranth may explain why ABA signaling and ER protein quality control pathways emerged as particularly important in our analysis, as these pathways are known to be critical for synthesis and accumulation of globulin-type storage proteins.

Future research should focus on fine-mapping the identified QTL to resolve the causal polymorphisms and validate the candidate genes through functional studies. The tandem gene duplications observed at multiple MTAs (NCED genes, bZIP transcription factors, ALFIN-like chromatin regulators) suggest that gene dosage effects or subfunctionalization may contribute to phenotypic variation, warranting investigation through gene expression analysis and allelic diversity studies. Several high-priority candidate genes also represent potential targets for genome editing or transgenic approaches to further enhance protein content, including overexpression of NCED genes to increase ABA levels, engineering of ABF2/AREB1 to create constitutively active forms, or modulation of KDEL receptor expression to fine-tune ER chaperone homeostasis. However, such approaches would require careful optimization to avoid pleiotropic effects, as these genes function in multiple developmental and physiological processes beyond storage protein accumulation.

## 5 Conclusion

This study establishes the first comprehensive genomic framework for seed protein composition in grain amaranth through multi-environment genome-wide association analysis of 192 globally diverse accessions. Systematic phenotyping of albumin, globulin, glutelin, prolamin, and total protein content across contrasting agro-ecological zones, combined with high-density genotyping (41,931 SNPs) and multi-locus GWAS, successfully mapped the genetic architecture underlying this nutritionally critical trait complex. Exceptionally high broad-sense heritability for albumin (H^2^ = 0.933) and total protein (H^2^ = 0.910), coupled with substantial genetic advance potential (60.78% for albumin), confirms these traits are highly amenable to genetic improvement through selection.

Rigorous cross-environmental validation identified 17 environmentally stable marker-trait associations for total protein, albumin, glutelin, and globulin, providing robust molecular tools deployable across diverse production environments. The absence of stable associations for prolamin confirms environment-specific genetic control, underscoring the necessity of multi-environment trials for complex nutritional traits. Nine pleiotropic loci exhibited universal synergistic effects, enabling efficient allele pyramiding for cumulative protein improvement without antagonistic trade-offs.

The most significant biological insight is the complete absence of structural seed storage protein genes among all 17 stable marker-trait associations. Despite surveying 48 candidate genes within 250 kb windows, no 11S globulins, 7S globulins, 2S albumins, or glutelins were detected, definitively establishing that natural variation operates entirely through trans-regulatory mechanisms. Functional enrichment analysis validated this architecture, identifying “histone binding” as the most significantly enriched molecular function (FDR = 0.035) and “aminoacyl-tRNA biosynthesis” as the sole significantly enriched KEGG pathway (FDR = 0.020), demonstrating coordinated control through chromatin accessibility, translation capacity, ER protein quality control, and hormone signaling bottlenecks.

Candidate gene analysis delineated four major regulatory modules: (1) a complete ABA biosynthesis-to-signaling cascade for glutelin, comprising NCED enzymes and ABF2/AREB1 transcription factors across independent loci; (2) ER protein folding capacity limiting globulin through KDEL receptor and DnaJ/Hsp40 chaperones; (3) translation machinery and amino acid transport constraining total protein via aminoacyl-tRNA synthetases and BAT1 transporter; and (4) chromatin-level regulation through ALFIN-like PHD finger proteins and ATX1 histone methyltransferase coordinating multiple biosynthetic pathways. The trans-regulatory nature of all identified loci is particularly advantageous for breeding, as selection on regulatory variants can enhance protein content while preserving amaranth’s nutritionally superior albumin and globulin composition.

This research provides immediate tools for precision breeding through environmentally stable markers and validated biological pathways. Integration of these markers into marker-assisted selection programs, combined with phenotypic selection for high-heritability traits, will accelerate development of nutritionally superior amaranth cultivars with broad environmental adaptability, advancing this climate-resilient pseudocereal’s contribution to global food security and malnutrition mitigation.

## Data Availability

The datasets presented in this study can be found in online repositories. All raw sequencing data are accessible through the NCBI Sequence Read Archive under accession number PRJNA1208757.
