# Supplementary material for: Genetic architecture of seed protein composition in grain amaranth (Amaranthus hypochondriacus): a multi-environment genome-wide association study
Source: Front Nutr. 2026 Mar 10;13:1758193. doi: 10.3389/fnut.2026.1758193 (PMC13008625; doi:10.3389/fnut.2026.1758193)
Supplement: Supplementary file 1 [file Table_1.docx]

**Supplementary Table : Germplasm characteristics**


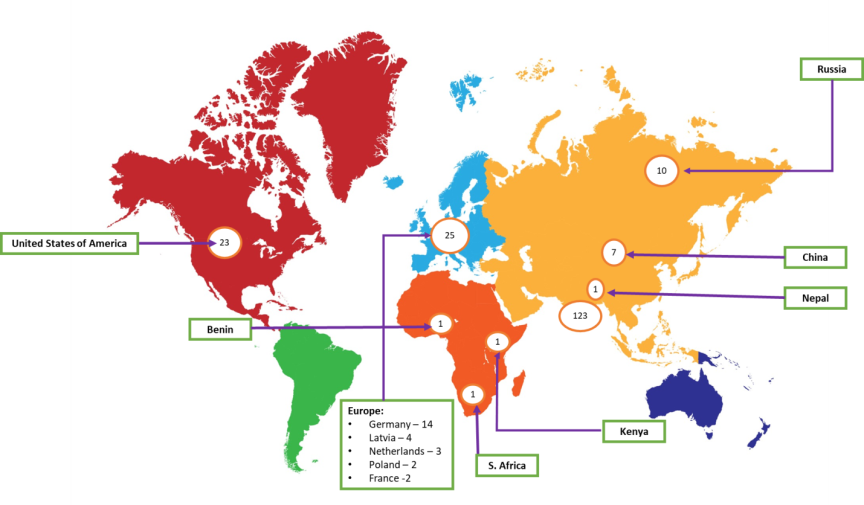


| **S.No.** | **Accessions** | **Geographical Origin** |
| --- | --- | --- |
| 1 | IC-35421 | Anjangaon, Amravati, Maharashtra |
| 2 | IC-35422 | Anjangaon, Amravati, Maharashtra |
| 3 | IC-35423 | Anjangaon, Amravati, Maharashtra |
| 4 | IC-35424 | Anjangaon, Amravati, Maharashtra |
| 5 | IC-35428 | Anjangaon, Amravati, Maharashtra |
| 6 | IC-35430 | Anjangaon, Amravati, Maharashtra |
| 7 | IC-35431 | Anjangaon, Amravati, Maharashtra |
| 8 | IC-35492 | Karasgaon, Amravati, Maharashtra |
| 9 | IC-35493 | Nagpur, Maharashtra |
| 10 | IC-35361 | Patur, Balapur, Akola, Maharashtra |
| 11 | IC-35362 | Patur, Balapur, Akola, Maharashtra |
| 12 | IC-35367 | Patur, Balapur, Akola, Maharashtra |
| 13 | IC-35368 | Patur, Balapur, Akola, Maharashtra |
| 14 | IC-35370 | Patur, Balapur, Akola, Maharashtra |
| 15 | IC-35371 | Patur, Balapur, Akola, Maharashtra |
| 16 | IC-35372 | Patur, Balapur, Akola, Maharashtra |
| 17 | IC-35374 | Patur, Balapur, Akola, Maharashtra |
| 18 | IC-35375 | Patur, Balapur, Akola, Maharashtra |
| 19 | IC-35377 | Patur, Balapur, Akola, Maharashtra |
| 20 | IC-35378 | Patur, Balapur, Akola, Maharashtra |
| 21 | IC-35383 | Patur, Balapur, Akola, Maharashtra |
| 22 | IC-35414 | Anjangaon, Amravati, Maharashtra |
| 23 | IC-35416 | Anjangaon, Amravati, Maharashtra |
| 24 | IC-35419 | Anjangaon, Amravati, Maharashtra |
| 25 | IC-35420 | Anjangaon, Amravati, Maharashtra |
| 26 | IC- 35376 | Patur, Balapur, Akola, Maharashtra |
| 27 | IC-35417 | Anjangaon, Amravati, Maharashtra |
| 28 | IC-35363 | Patur, Balapur, Akola, Maharashtra |
| 29 | IC-35364 | Patur, Balapur, Akola, Maharashtra |
| 30 | IC-35413 | Anjangaon, Amravati, Maharashtra |
| 31 | IC-35432 | Anjangaon, Amravati, Maharashtra |
| 32 | IC-35381 | Patur, Balapur, Akola, Maharashtra |
| 33 | IC-35426 | Anjangaon, Amravati, Maharashtra |
| 34 | IC-35380 | Patur, Balapur, Akola, Maharashtra |
| 35 | IC-35379 | Patur, Balapur, Akola, Maharashtra |
| 36 | IC-35373 | Patur, Balapur, Akola, Maharashtra |
| 37 | IC-35369 | Patur, Balapur, Akola, Maharashtra |
| 38 | IC-26264 | Akola, Maharashtra |
| 39 | IC-35366 | Patur, Balapur, Akola, Maharashtra |
| 40 | IC-35382 | Patur, Balapur, Akola, Maharashtra |
| 41 | IC-35365 | Patur,Balapur,AKOLA,Maharashtra |
| 42 | IC-35415 | Anjangaon,,Amravati ,Maharashtra |
| 43 | IC-467910 | Kurthla, Chamba, Himachal Pradesh |
| 44 | IC-36831 | Panjain, Mandi, Himachal Pradesh |
| 45 | IC-36833 | Bago, Shimla, Himachal Pradesh |
| 46 | IC-36834 | Kathog, Shimla, Himachal Pradesh |
| 47 | IC-38039 | Shimla, Himachal Pradesh |
| 48 | IC-38054 | Shimla, Himachal Pradesh |
| 49 | IC-38160 | Mandi, Himachal Pradesh |
| 50 | IC-38161 | Mandi, Himachal Pradesh |
| 51 | IC-38192 | Mandi, Himachal Pradesh |
| 52 | IC-38193 | Mandi, Himachal Pradesh |
| 53 | IC- 17935 | Jeori, Shimla, Himachal Pradesh |
| 54 | IC-17936 | Narkanda, Shimla, Himachal Pradesh |
| 55 | IC-36835 | Sariwan,, Shimla, Himachal Pradesh |
| 56 | IC-38162 | Mandi, Himachal Pradesh |
| 57 | IC-38057 | Shimla, Himachal Pradesh |
| 58 | IC-36830 | Mangoli,Mandi, Himachal Pradesh |
| 59 | IC-329587 | Tindi Udaipur, Lahaul&Spiti, Himachal Pradesh |
| 60 | IC-36832 | Moag, Shimla, Himachal Pradesh |
| 61 | IC-38055 | Shimla, Himachal Pradesh |
| 62 | IC-38163 | Mandi, Himachal Pradesh |
| 63 | IC-38164 | Mandi, Himachal Pradesh |
| 64 | IC-38056 | Shimla, Himachal Pradesh |
| 65 | IC-38166 | Mandi, Himachal Pradesh |
| 66 | IC - 38058 | Shimla, Himachal Pradesh |
| 67 | IC-106354 | Chackloh,Hamirpur, Himachal Pradesh |
| 68 | IC-329588 | Tindi Udaipur, Lahaul&Spiti, Himachal Pradesh |
| 69 | IC-38159 | Mandi, Himachal Pradesh |
| 70 | IC-38065 | Shimla, Himachal Pradesh |
| 71 | IC-547510 | Jwasa,, Uttarkashi, Uttarakhand |
| 72 | IC-547511 | Paughat,, Uttarkashi, Uttarakhand |
| 73 | IC-582932 | Atala,Utarkashi, Uttarakhand |
| 74 | IC-582935 | Atala, Utarkashi, Uttarakhand |
| 75 | IC-47436 | Narayan Ashram, Pithoragarh, Uttarakhand |
| 76 | IC-47437 | Narayan Ashram, Pithoragarh , Uttarakhand |
| 77 | IC-24263 | Sama,, Almora, Uttarakhand |
| 78 | IC-355789 | Palvana, Almora, Uttarakhand |
| 79 | IC-24264 | Naehti, Loharkhot, Almora, Uttarakhand |
| 80 | IC-24266 | Mollikhal, Salt block, Bageshwar, Uttarakhand |
| 81 | IC-24265 | Saling,Loharkhot,Bageshwar,Uttarakhand |
| 82 | IC-264790 | Bhatraj, Nainital, Uttarakhand |
| 83 | IC-41997 | Mulai, Betul, Madhya Pradesh |
| 84 | IC-41998 | Ambada,, Betul, Madhya Pradesh |
| 85 | IC-41999 | Ambada, Betul, Madhya Pradesh |
| 86 | IC-42000 | Pagara near Pachmarhi,, Hoshangabad, Madhya Pradesh |
| 87 | IC-21795 | Odagi, Singrauli, Madhya Pradesh |
| 88 | IC-21804 | Karangia, Dindori, Madhya Pradesh |
| 89 | IC-21938 | Shahpura, Dindori, Madhya Pradesh |
| 90 | IC-21808 | Odagi,Singrauli, Madhya Pradesh |
| 91 | IC-21931 | Kasturi,Dindori, Madhya Pradesh |
| 92 | IC-42001 | Pagara near Pachmarhi, Hoshangabad, Madhya Pradesh |
| 93 | IC41996 | Multai,Betul, Madhya Pradesh |
| 94 | IC21930 | Bichhiya, Vidisha, Madhya Pradesh |
| 95 | IC-41769 | Ligtam, East Sikkim, Sikkim |
| 96 | IC-41767 | S. Rigu,, East Sikkim, Sikkim |
| 97 | IC-41768 | Khechepessi, west Sikkim, Sikkim |
| 98 | IC-21947 | Gharghoda,, Raigarh, Sikkim |
| 99 | IC-21961 | Marwahi, Bilaspur, Sikkim |
| 100 | IC-21967 | Gharghoda, Raigarh, Sikkim |
| 101 | IC-21973 | Raigarh, Sikkim |
| 102 | IC-21960 | Marwahi, Bilaspur, Sikkim |
| 103 | IC-35774 | Isaran, Ahmdabad, Gujarat |
| 104 | IC-35780 | Isaran, Ahmdabad, Gujarat |
| 105 | IC-35777 | Isaran, Ahmadabad, Gujarat |
| 106 | IC-35775 | Isaran, Ahmadabad, Gujarat |
| 107 | IC-35776 | Isaran,Ahmadabad, Gujarat |
| 108 | IC-35770 | Isaran, Ahmadabad, Gujarat |
| 109 | IC-35520 | Salai,Mehsana ,Gujarat |
| 110 | IC -20303 | Kruin, Mawryngkneng,,East Khasi Hills, Meghalaya |
| 111 | IC-20304 | Puringmawryngkneng, East Khasi Hills, Meghalaya |
| 112 | IC-20306 | Zigzag, East Garo Hills, Meghalaya |
| 113 | IC-258370 | Norbugh, Badgam, Jammu & Kashmir |
| 114 | IC-361853 | Thakur, Kapran,, Anantnag, Jammu & Kashmir |
| 115 | IC-328559 | Bani, Bashli, Kathua, Jammu & Kashmir |
| 116 | IC-26270 | Malkapur, BIDAR, Karnataka |
| 117 | IC-26266 | Bijapur, Bijapur, Karnataka |
| 118 | IC-26272 | Nanora, Bicholim, North Goa, Goa |
| 119 | IC-396983 | Ladpura,, Bhilwara, Rajasthan |
| 120 | IC-21939 | Tapkara,Raigarh, Chattisgarh |
| 121 | NAIP 42 | Unknown |
| 122 | NAIP 46 | Unknown |
| 123 | NAIP 49 | Unknown |
| 124 | EC-338765 | United States Of America |
| 125 | EC-146535 | United States Of America |
| 126 | EC-146508 | United States Of America |
| 127 | EC-146537 | United States Of America |
| 128 | EC-146541 | United States Of America |
| 129 | EC-146536 | United States Of America |
| 130 | EC-146506 | United States Of America |
| 131 | EC-146510 | United States Of America |
| 132 | EC-146509 | United States Of America |
| 133 | EC-146539 | United States Of America |
| 134 | EC- 146543 | United States Of America |
| 135 | EC-146493 | United States Of America |
| 136 | EC-146494 | United States Of America |
| 137 | EC-146533 | United States Of America |
| 138 | EC-146538 | United States Of America |
| 139 | EC-146542 | United States Of America |
| 140 | EC-146532 | United States Of America |
| 141 | EC-146540 | United States Of America |
| 142 | EC-146507 | United States Of America |
| 143 | EC145973 | United States Of America |
| 144 | EC146534 | United States Of America |
| 145 | EC-289386 | United States Of America |
| 146 | EC-289393 | United States Of America |
| 147 | EC-328885 | Germany |
| 148 | EC328875 | Germany |
| 149 | EC-328873 | Germany |
| 150 | EC-328896 | Germany |
| 151 | EC-328883 | Germany |
| 152 | EC-328881 | Germany |
| 153 | EC-328880 | Germany |
| 154 | EC-328878 | Germany |
| 155 | EC-328877 | Germany |
| 156 | EC-328874 | Germany |
| 157 | EC-328879 | Germany |
| 158 | EC-328876 | Germany |
| 159 | EC-328886 | Germany |
| 160 | EC-328891 | Germany |
| 161 | ES- 519526 | Russia |
| 162 | EC519523 | Russia |
| 163 | EC-519527 | Russia |
| 164 | EC-519512 | Russia |
| 165 | EC-519511 | Russia |
| 166 | EC-519510 | Russia |
| 167 | EC-519509 | Russia |
| 168 | EC-519541 | Russia |
| 169 | EC-519542 | Russia |
| 170 | EC-519544 | Russia |
| 171 | EC-345800 | China |
| 172 | EC-120051 | China |
| 173 | EC-120048 | China |
| 174 | EC-120049 | China |
| 175 | EC-120052 | China |
| 176 | EC-120050 | China |
| 177 | EC-345797 | China |
| 178 | EC-333744 | Latvia |
| 179 | EC-333747 | Latvia |
| 180 | EC333745 | Latvia |
| 181 | EC333748 | Latvia |
| 182 | EC-150190 | Netherlands |
| 183 | EC-150191 | Netherlands |
| 184 | EC-150192 | Netherlands |
| 185 | EC-32881 | France |
| 186 | EC-351945 | France |
| 187 | EC-157314 | Poland |
| 188 | EC-157313 | Poland |
| 189 | EC-18862 | Nepal |
| 190 | EC-151544 | Benin |
| 191 | EC-223672 | Kenya |
| 192 | EC-583624 | South Africa |
