# Supplementary material for: Genetic architecture of seed protein composition in grain amaranth (Amaranthus hypochondriacus): a multi-environment genome-wide association study
Source: Front Nutr. 2026 Mar 10;13:1758193. doi: 10.3389/fnut.2026.1758193 (PMC13008625; doi:10.3389/fnut.2026.1758193)
Supplement: Supplementary file 2 [file Table_2.docx]

**Table: Summary of Geographical and Environmental characteristics of the two experimental sites**

| **Parameter** | **VPKAS-Almora** | **PCPGR-Pantnagar** |
| --- | --- | --- |
| Latitude (°N) | 29.589 | 29.03 |
| Longitude (°E) | 79.647 | 79.46 |
| Altitude (m AMSL) | 1600 | 244 |
| Agro-ecological zone | Western Himalayan hill region | Tarai / Indo-Gangetic plains |
| Climate classification | Humid subtropical (monsoon-influenced, cooler hills) | Humid subtropical (monsoon-influenced plains) |
| Growing season | June–November 2022 | June–November 2021 |
| Mean temperatures (°C) (Jun–Nov) | 28.5 (Max), 15.4 (Min) Range: 4.4 - 33.1 | 31.8 (Max), 22.3 (Min) Range: 12.1 - 37.0 |
| Mean relative humidity (%) (Jun–Nov) | 85.5% (I hour), 57.2% (II hour) | 86.7% (I hour), 58.8% (II hour) |
| Total no. of rainy days (Jun–Nov) | 45 | 36 |
| Mean sunshine hours (Jun–Nov) | 6.40 hrs/day | 6.1 hrs/day |
| Mean wind speed (Jun–Nov) | 1.6 km/h | 3.8 km/h |
| Precipitation pattern | High monsoon rainfall (June–September) with peak in July–August; low post-monsoon rainfall | High monsoon rainfall with peak in July–August; declining rainfall in October–November |
| Estimated total precipitation (Jun–Nov, mm) | ~565 (majority during monsoon months) | ~1138 (majority during monsoon months) |
| Growing conditions | Cooler temperatures, hilly terrain, high monsoon rainfall, rapid post-monsoon drying | Warm temperatures, flat alluvial soils, high monsoon rainfall, gradual post-monsoon drying |
