# Supplementary material for: Genetic architecture of seed protein composition in grain amaranth (Amaranthus hypochondriacus): a multi-environment genome-wide association study
Source: Front Nutr. 2026 Mar 10;13:1758193. doi: 10.3389/fnut.2026.1758193 (PMC13008625; doi:10.3389/fnut.2026.1758193)
Supplement: Supplementary file 6 [file Image_1.pdf]

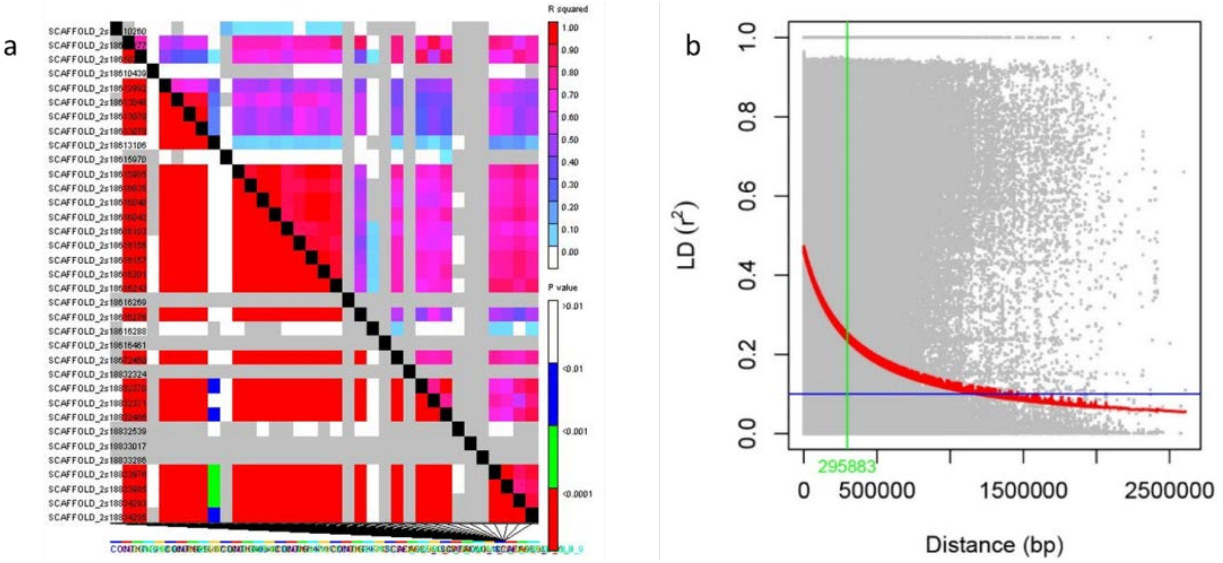

Supplementary figure: Linkage Disequilibrium Plot. Markers were ordered on the x- and y-axes based on genomic location; hence, every cell of the heat map represents a single marker pair. (b) **Linkage disequilibrium decay curve** based on 41,931 polymorphic SNPs in 192 *A. hypochondriacus* accessions. The red curve indicates the general trend in LD decay. The vertical (green) line signifies the LD decay distance, while the horizontal line (dark blue) represents the half LD values.
